# Molecular Docking and Molecular Dynamics Studies Reveal Secretory Proteins as Novel Targets of Temozolomide in Glioblastoma Multiforme

**DOI:** 10.3390/molecules27217198

**Published:** 2022-10-24

**Authors:** Sumera, Farha Anwer, Maaz Waseem, Areeba Fatima, Nishat Malik, Amjad Ali, Saadia Zahid

**Affiliations:** 1 Neurobiology Research Laboratory, Department of Healthcare Biotechnology, Atta-ur-Rahman School of Applied Biosciences (ASAB), National University of Sciences and Technology (NUST), Islamabad 44000, Pakistan; 2 Integrative Biology Laboratory, Department of Industrial Biotechnology, Atta-ur-Rahman School of Applied Biosciences (ASAB), National University of Sciences and Technology (NUST), Islamabad 44000, Pakistan

**Keywords:** glioblastoma multiforme, temozolomide, molecular dynamics simulation, molecular docking, drug resistance

## Abstract

Glioblastoma multiforme (GBM) is a tumor of glial origin and is the most malignant, aggressive and prevalent type, with the highest mortality rate in adult brain cancer. Surgical resection of the tumor followed by Temozolomide (TMZ) therapy is currently available, but the development of resistance to TMZ is a common limiting factor in effective treatment. The present study investigated the potential interactions of TMZ with several secretory proteins involved in various molecular and cellular processes in GBM. Automated docking studies were performed using AutoDock 4.2, which showed an encouraging binding affinity of TMZ towards all targeted proteins, with the strongest interaction and binding affinity with GDF1 and SLIT1, followed by NPTX1, CREG2 and SERPINI, among the selected proteins. Molecular dynamics (MD) simulations of protein–ligand complexes were performed via CABS-flex V2.0 and the iMOD server to evaluate the root-mean-square fluctuations (RMSFs) and measure protein stability, respectively. The results showed that docked models were more flexible and stable with TMZ, suggesting that it may be able to target putative proteins implicated in gliomagenesis that may impact radioresistance. However, additional in vitro and in vivo investigations can ascertain the potential of the selected proteins to serve as novel targets for TMZ for GBM treatment.

## 1. Introduction

**Figure 1:**
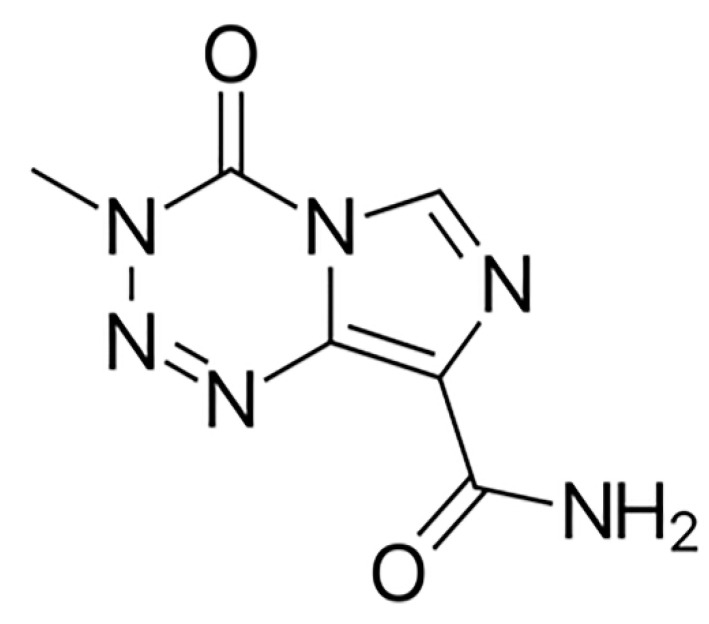
Chemical structure of Temozolomide, acquired from PubChem database.

Glioblastoma multiforme (GBM) is a primary malignant tumor of the brain and encompasses 16% of all primary neoplasms of the brain and CNS [1]. It is the most common among primary malignant brain tumors, with an average age-adjusted incidence rate of 3.2 per 100,000 population [2,3]. Although GBM can present at any age, it is more prevalent at a median age of 64 years [1]. The incidence is found to be higher in men in comparison to women (1.6:1) [4]. GBM is classified as primary, which arises spontaneously without a known precursor, or secondary, which develops gradually from a low-grade tumor. Primary GBM constitutes the majority of these neoplasms occurring in older patients and exhibits poorer prognoses as compared to patients with secondary GBM [5]. Therapeutic interventions consist of Temozolomide (TMZ), which is given as the first line of therapy following surgery and also as an adjuvant drug in combination with radiation therapy. It is a DNA-alkylating agent that targets guanine at the O6 and N7 positions (Figure 1) and exerts its therapeutic effect by inducing cancer cell apoptosis and preventing DNA replication [6].

Currently, GBM is diagnosed through imaging techniques and tissue biopsies [7]. However, there are various constraints associated with these techniques. The drawback of imaging techniques is that they cannot differentiate lesions induced by actual tumor progression from those occurring due to treatment-associated pseudo-progression that resolve naturally with time. Likewise, tissue biopsies present the disadvantage of being highly invasive and providing a static snapshot of the constantly evolving tumor. In contrast, liquid biopsies have emerged as being more favorable techniques due to their ability to detect circulating biomarkers, along with being non-invasive, thus allowing for serial sampling to screen dynamic modifications in the tumor during the course of therapy [8,9,10].

Generally, tumors, including GBM, release tumor-associated contents, such as proteins, extracellular vehicles (EVs), circulating tumor cells (CTCs) and cell-free nucleic acids (cfNAs), into the blood [11] and cerebrospinal fluid (CSF) [12]. In GBM, the mechanisms behind extraneural metastasis are lymphatic spread, direct proliferation through the dura and bone and via venous invasion [13]. Secreted biomarkers can be detected in liquid biopsies and serve to complement standard risk-stratification approaches, observing the response to treatment and the progression of the disease in GBM patients [14]. The nine proteins selected in this study are brain-enriched secretory proteins that are differentially regulated in GBM and involved in oncogenic pathways involved in GBM development, i.e., opioid binding protein/cell adhesion molecule-like (OPCML), neuronal pentraxin 1 (NPTX1), contactin 2 (CNTN2), leucine-rich glioma-inactivated 1 (LGI1), lymphocyte antigen 6 family member H (LY6H), slit guidance ligand 1 (SLIT1), growth differentiation factor 1 (GDF1), cellular repressor of E1A-stimulated genes 2 (CREG2) and serine proteinase inhibitor 1 (SERPINI1).

CNTN2 is a brain-enriched secretory protein detected in circulating exosomes [15]. The protein expression level of CNTN2 is upregulated in human glioma cells as compared to normal human astrocytes [16]. It substantially promotes the proliferation of U87-GSCs through the activation of APP/AICD, Notch/HES1 and EGFR/PI3K/AKT pathways, suggesting that it is an absolute upstream target for the development of glioma therapeutics [17]. Similarly, NPTX1, another secreted protein [18], promotes tumor development and mediates cell proliferation and differentiation through the regulation of the IRS1/PI3K/AKT pathway; however, it can be inhibited by miR-128-3p [19]. Interestingly, several long noncoding RNAs, for example, SLC26A4-AS1, upregulate NPTX1 through the NFKB1 transcription factor to produce antiangiogenic effects in glioma cells [20]. The downregulation of OPCML, which is secreted in CSF, was also observed in GBM cells and tissue [21], while CREG2, a secreted glycoprotein explicitly expressed in the brain, is upregulated in GBM compared to normal brain tissue [22,23].

GDF1 is a secreted glycoprotein and a member of the transforming growth factor-β (TGF-β) superfamily [24]. A supervised clustering analysis of 43 genes showed that GDF1 is highly expressed in brain cancers, including GBM [25]. In contrast, LGI1, which is also a secreted neuronal protein, has markedly negligible expression in GBM compared to normal brain tissue [26,27]. LGI1 utilizes the phosphatidylinositol 3-kinase/ERK pathway and suppresses the production of MMP1/3; therefore, the loss of its expression results in the invasive phenotype in GBM [28]. Likewise, LY6H, another secretory protein, is highly expressed in GBM tissues compared to the normal brain [29]. We also considered the SERPINI1 protein, which is principally secreted by neurons. A remarkable reduction in its expression was observed in the peritumoral tissues of GBM compared to controls [30]. Lastly, SLIT1, which is a secreted axon guidance molecule [31], has substantially downregulated protein and mRNA expression in GBM tissues compared to normal tissues. It is significantly suppressed by miR-640 and consequently stimulates the adhesion and proliferation of GBM cell lines, suggesting that the miR-640/SLIT1 axis might have the potential to act as a novel target for the treatment and diagnosis of GBM [32].

The current study evaluated the plausible interactions of these targeted proteins to provide baseline data highlighting the selected proteins as possible therapeutic targets of TMZ, which can further lead to the elucidation of molecular and cellular processes involved in GBM. The selected proteins were initially docked via AutoDock 4.0 to examine their binding affinity with TMZ, and for the further assessment of the flexibility and stability of the TMZ–protein complexes, molecular dynamics (MD) simulations utilizing CABS-flex and iMODS were performed.

## 2. Results

### 2.1. Potential Binding Affinity of TMZ towards the Nine Proteins

**Figure 2:**
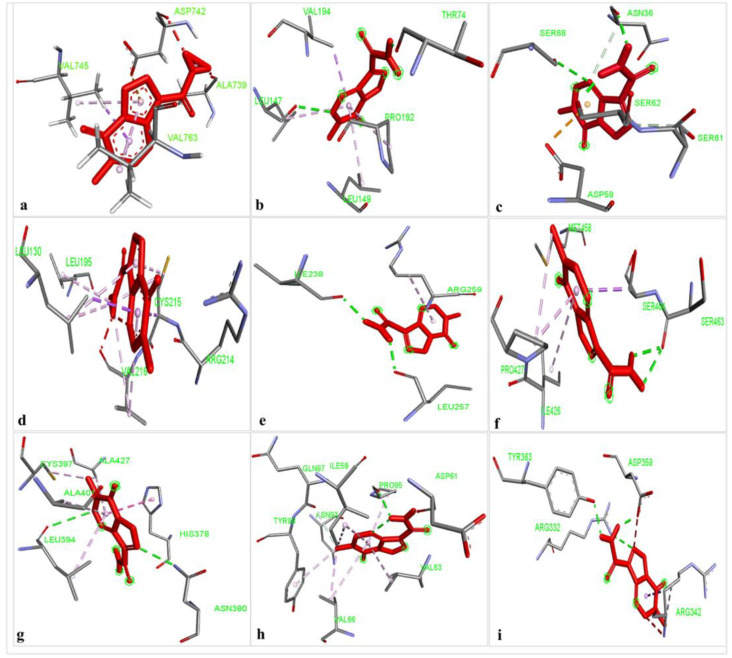
The identification of non-covalent interactions between TMZ and (**a**) SLIT1; (**b**) GDF1; (**c**) LY6H; (**d**) CREG2; (**e**) SERPIN1; (**f**) LGI1; (**g**) NPTX1; (**h**) OPCML; and (**i**) CNTN2.

**Figure 3:**
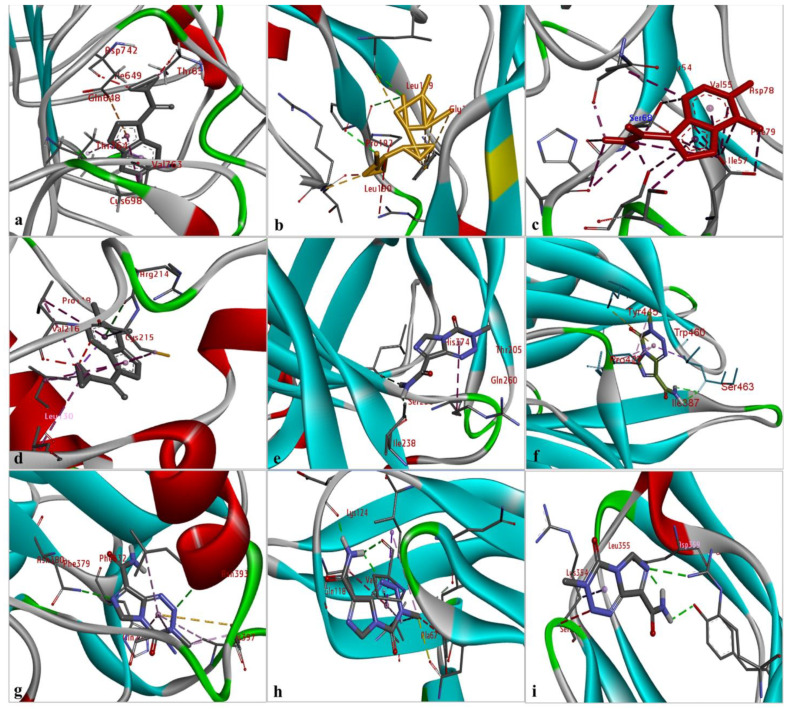
The identification of the peptide conformations of interacting residues of the selected proteins: (**a**) SLIT1; (**b**) GDF1; (**c**) LY6H; (**d**) CREG2; (**e**) SERPIN1; (**f**) LGI1; (**g**) NPTX1; (**h**) OPCML; and (**i**) CNTN2.

The results of the comparative analysis revealed that SLIT1 and GDF1 had the highest binding affinity for TMZ, represented by the lowest binding energies of −9.95 and −9.87 Kcal/mol, respectively. Nevertheless, GDF1 formed two hydrogen bonds (LEU147 and THR74) along with other interactions, but SLIT1 did not show any hydrogen bonding while interacting with TMZ. However, SLIT1 has significant binding energy based on other interactions (Figure 2 and Table 1). The peptide conformation of SLIT1 revealed that the maximum binding of TMZ occurred with residues ASP742 and VAL745, which were detected in the coiled region of the protein. A peptide conformation similar to that of SLIT1 was observed in GDF1; the residues interacting with TMZ were detected in the β-sheet conformation of the protein (Figure 3).

Similarly, NPTX1 and SERPINI1 formed two hydrogen bonds with TMZ, but CREG2 had no hydrogen bonds. However, the binding potential towards TMZ was similar in these three proteins, as represented by binding energies of −8.92, −8.06 and −8.73 Kcal/mol, respectively (Figure 2 and Table 1). The peptide conformations of the interacting residues of NPTX1 and SERPINI1 existed in the β-pleated sheets of both proteins, whereas the interacting residues of CREG2 were in the coiled (CYS215, VAL216 and ARG214) and α-helical (LEU120) regions of the protein structure (Figure 3).

OPCML and LGI1 showed comparatively lower binding affinities of −7.81 and −7.26 Kcal/mol, respectively, for TMZ and formed two hydrogen bonds, along with other interactions (Figure 2 and Table 1). The peptide conformation of the interacting residues of both of these proteins existed in their β-pleated regions (Figure 3). CNTN2 and LY6H showed the lowest binding affinities, with binding energies of −6.92 and −6.55 Kcal/mol, respectively. However, CNTN2 and LY6H interacted with TMZ through three hydrogen bonds, which is the highest number of hydrogen bonds in comparison to the other proteins (Figure 2 and Table 1). The peptide conformation of the interacting residues of LY6H existed in β-pleated (SER62) and coiled (ASP59 and SER68) regions, while the peptide conformation of CNTN2 was detected to be both α-helical (ASP359) and coiled (ARG342) (Figure 3).

### 2.2. Molecular Dynamics Simulation

**Figure 4:**
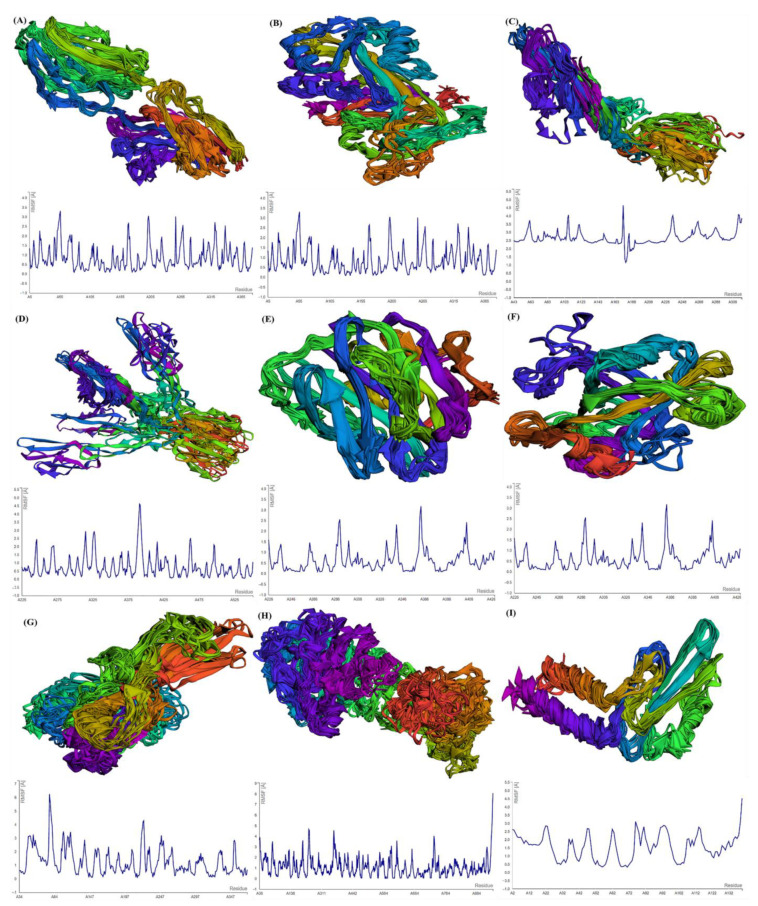
Multimodel superimposed simulated structure and MD simulation showing the RMSF profiles of (**A**) SLIT1; (**B**) CNTN2; (**C**) OPCML; (**D**) LGI1; (**E**) NPTX1; (**F**) CREG2; (**G**) GDF1; (**H**) SLIT1; and (**I**) LY6H.

For the estimation of protein–ligand complex stability, MD simulations were performed. MD simulations are able to assess ligand-induced alterations in the protein structure. The RMSF profiles of selected GBM proteins generated using CABS-flex show the flexibility of the amino acids (Figure 4). The maximum RMSF value reveals more flexibility, whereas the minimum value suggests the limited motion of the system throughout the simulation course.

After submitting the protein structure (in PDB format with the default parameters) to CABS-flex, the server generates an output file including 10 modeled structures and the root-mean-square fluctuation (RMSF) profile in a graph to calculate, per residue, fluctuations in the protein/peptide complex (Figure 4). CNTN2 showed the maximum fluctuation (3.365 Å) at residue no. 4 and the minimum fluctuation (0.081) at residue no. 138 of chain A. For SERPINI1, the maximum fluctuation (8.807 Å) was observed at residue no. 353, whereas the minimum fluctuation (0.059 Å) was observed at residue no. 342 of chain A. OPCML showed the maximum fluctuation (4.058 Å) at residue no. 108 and the minimum fluctuation (1.158 Å) at residue no. 181 of chain A. LGI1 displayed the maximum fluctuation (Max: 4.614 Å) at residue no. 391 and the minimum fluctuation (0.052 Å) at residue no. 525 of chain A. For NPTX1, the maximum fluctuation (Max: 3.185 Å) was observed at residue no. 225, and the minimum fluctuation (0.087 Å) was observed at residue no. 272 of chain A. CREG2 demonstrated the maximum fluctuation (6.242 Å) at residue no. 77, while the minimum fluctuation (0.107 Å) was observed at residue no. 220. GDF1 exhibited the maximum fluctuation (6.242 Å) at residue no. 77, whereas the minimum fluctuation (0.107 Å) occurred at residue no. 150. LY6H presented the maximum fluctuation (6.196 Å) at residue no. 53, whereas the minimum fluctuation (0.2 Å) occurred at residue no. 94. SLIT1 showed the maximum fluctuation (8.078 Å) at residue no. 917, whereas the minimum fluctuation (0.044 Å) was observed at residue no. 577.

The complete residue fluctuation profiles for the nine proteins, i.e., CNTN2, SERPINI1, OPCML, LGI1, NPTX1, CREG2, GDF1, LY6H and SLIT1, are given in Supplementary Materials Tables S1–S9, representing the relative propensities of protein residues to diverge from the average dynamic structure.

**Figure 5:**
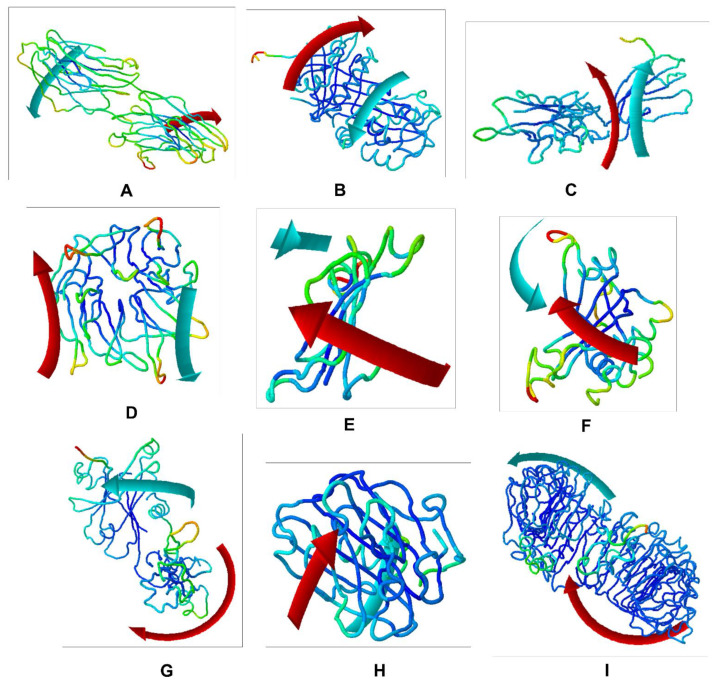
Molecular mobility evaluated by NMA of the docked complexes: (**A**) CNTN2-TMZ; (**B**) SERPINI1-TMZ; (**C**) OPCML-TMZ; (**D**) LGI1-TMZ; (**E**) NPTX1-TMZ; (**F**) CREG2-TMZ; (**G**) GDF1-TMZ; (**H**) LY6H-TMZ; and (**I**) SLIT1-TMZ. The two colored affine arrows display the mobility or the direction of motion, where the longer arrows indicate greater motion.

To evaluate the stability and physical movements of the docked complexes, we performed MD simulations using the iMOD server. Normal mode analysis (NMA) was implemented to investigate the slow dynamics of the docked complexes and to demonstrate their large-amplitude conformational fluctuations. NMA of the docked complexes, i.e., CNTN2-TMZ, SERPINI1-TMZ, OPCML-TMZ, LGI1-TMZ, NPTX1-TMZ, CREG2-TMZ, GDF1-TMZ, LY6H-TMZ and SLIT1-TMZ, is illustrated in Figure 5.

**Figure 6:**
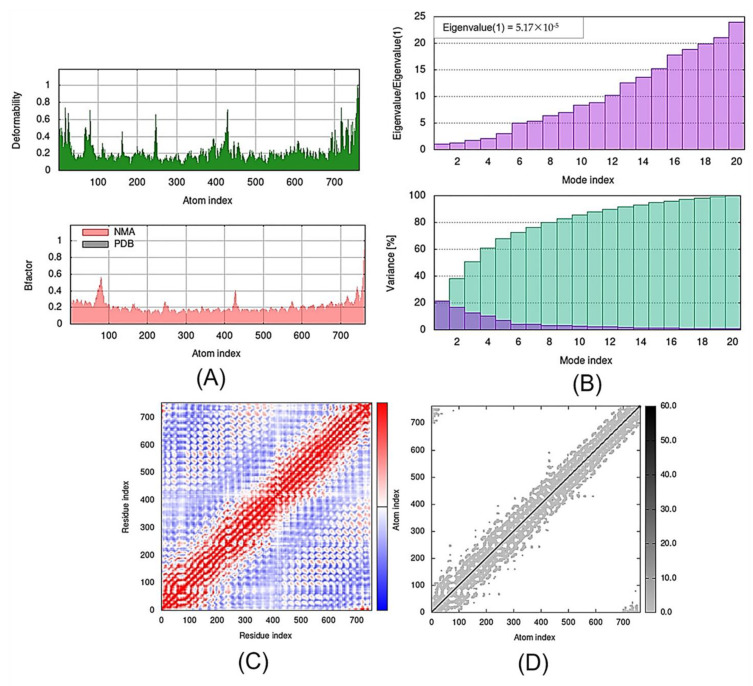
Outputs of molecular dynamics simulations in iMODS for SLIT1-TMZ: (**A**) deformability and B-factor plot; (**B**) eigenvalue and variance plot; (**C**) elastic network model; and (**D**) covariance map.

The deformability and B-factor give the mobility profiles of the docked proteins. The deformability and B-factors of the SLIT1-TMZ and GDF1-TMZ complexes illustrate the peaks corresponding to the regions in the proteins with deformability, where the highest peaks represent the regions of high deformability. The B-factor graphs provide a comparison between the NMA and the PDB field of the complexes (Figure 5 and Figure 6). The deformability and B-factor of NPTX1-TMZ, CREG2-TMZ, SERPINI1-TMZ, OPCML-TMZ, LGI1-TMZ, CNTN2-TMZ and LY6H-TMZ are illustrated in Supplementary Materials Figures S1–S7, respectively.

The eigenvalue and the variance are inversely linked to each normal mode. The eigenvalue and variance graphs of the SLIT1-TMZ and GDF1-TMZ complexes are depicted in Figure 5 and Figure 6, respectively. The variance graph of TMZ with the target proteins indicates the individual variance with purple-shaded bars, whereas cumulative variance is represented by green-shaded bars. The eigenvalue for each complex is provided in Table 2, whereas the eigenvalue and variance graphs of NPTX1-TMZ, CREG2-TMZ, SERPINI1-TMZ, OPCML-TMZ, LGI1-TMZ, CNTN2-TMZ and LY6H-TMZ are provided in Supplementary Materials Figures S1–S7, respectively.

**Figure 7:**
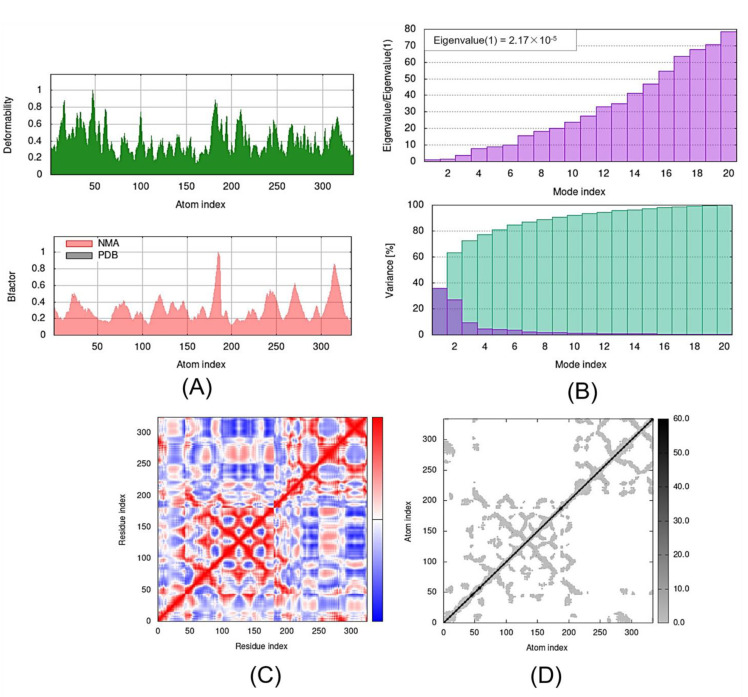
Outputs of molecular dynamics simulations through iMODS for GDF1-TMZ: (**A**) deformability and B-factor plot; (**B**) eigenvalue and variance plot; (**C**) elastic network model; and (**D**) covariance map.

The covariance matrix of the SLIT1-TMZ and GDF1-TMZ complexes displays the correlations among residues in a complex. The red color in the matrix illustrates a decent correlation between residues, whereas the white color indicates uncorrelated motion. Moreover, the blue color shows anticorrelations. The greater the correlation, the better the complex. The elastic maps of the docked proteins display the associations among the atoms, where the darker-gray portions specify stiffer portions (Figure 6 and Figure 7). The covariance matrices and elastic maps of NPTX1-TMZ, CREG2-TMZ, SERPINI1-TMZ, OPCML-TMZ, LGI1-TMZ, CNTN2-TMZ and LY6H-TMZ are given in Supplementary Materials Figures S1–S7. The elastic maps of all nine proteins produced reasonable results.

## 3. Discussion

Molecular docking is a powerful in silico structure-based method that enables the prediction of ligand–target interactions at a molecular level. The present study highlights the potential of TMZ to interact with the secretory proteins involved in the pathological processes of GBM through molecular docking and interaction studies. To probe for alternate diagnostic, tumor-monitoring and less invasive strategies, as well as identify novel target molecules of TMZ, we chose these nine proteins based on the available literature about their function and potential association with tumors. 

Among the nine proteins, SLIT1 showed the highest binding affinity with TMZ. Although SLIT1 displayed no hydrogen bonds with TMZ, it nonetheless presented other interactions with TMZ that play a vital role in increasing the overall binding energy. The peptide conformation of SLIT1 revealed the maximum binding of TMZ with residues ASP742 and VAL745, which were detected in the coiled region of the protein. GDF1 also presented top-notch binding energy, but it was slightly less than that of SLIT1. Unlike SLIT1, GDF1 exhibited two hydrogen bonds with TMZ (LEU147 and THR74), among other bonding interactions. A peptide conformation similar to that of SLIT1 was observed in GDF1; the residues interacting with TMZ were detected in the β-sheet conformation of the protein. The present study focused on GDF1 because it is closely linked to poor tumor differentiation [33], while SLIT1 possesses significant oncogenic potential and is responsible for the development and metastasis of various types of cancers [34]. Therefore, the strongest binding affinity of GDF1 and SLIT1 towards TMZ in the current study highlights these proteins as promising novel targets of TMZ.

Similarly, NPTX1 and SERPINI1 showed significant binding potential with TMZ, but it was lower than that of GDF1 and SLIT1. Both proteins showed two hydrogen bonds with TMZ, but NPTX1 displayed a greater number of other bond interactions compared to SERPINI1, which leads to the relatively higher binding potential of NPTX1 with TMZ than SERPINI1. The increased expression of NPTX1 is reported to suppress the pro-angiogenic ability of glioma cells [35]. Interestingly, an in silico analysis approach revealed NPTX1 to be an upregulated gene in GBM tissues in comparison to normal brain tissues [23]. Likewise, SERPINI1 is the most commonly mutated gene and critical for brain metastases, so it is important to mitigate its expression alterations, which have been observed in various studies [36]. The current findings reveal the strong binding affinity of NPTXI1 and SERPINI1 with TMZ, indicating the promising potential of these proteins as candidate drug targets for GBM.

CREG2, a secreted glycoprotein, did not show any hydrogen bonding but displayed different types of other interactions with TMZ. The peptide conformation also revealed strong interactions of CREG2 residues, predominantly in the coiled and α-helical regions of the protein structure. CREG2 showed significant binding with TMZ, and considering its involvement in various cancers [37,38], it can serve as a target protein for TMZ.

OPCML and LGI1 showed comparatively lower binding potential with TMZ; however, both of these proteins showed two hydrogen bonds along with other interactions with TMZ. Interestingly, the peptide conformation of the interacting residues of both of these proteins existed in their β-pleated regions. As strong tumor suppressors and brain-specific secretory proteins [39,40], both OPCML and LGI1 are worth further investigation in GBM, and the strong binding affinity with TMZ observed in the present work indicates their potential as novel targets for TMZ.

The increased expression of CNTN2 and its role in proliferation makes it a novel target of GBM therapy [41]. In addition, the upregulation of LY6H is associated with poor outcomes in terms of the patient survival rate, which makes it a novel biomarker of poor cancer prognosis and a therapeutic target for multiple cancers [29]. We observed the highest binding energies for CNTN2 and LY6H, which demonstrate their lowest affinity for TMZ; however, they stabilize their interaction with TMZ with the highest number of hydrogen bonds, along with other interactions.

Through the MD simulation assessment of these nine proteins via CABS-flex, we observed that the RMSF peaks for all of these proteins showed several regions with high flexibility. The highest RMSFs were observed for SERPINI1, SLIT1, GDF1 and LY6H. A higher value of RMSF demonstrates more flexible movements, whereas a low value of RMSF specifies restricted movements throughout the simulation from its average position [42]. CABS-flex obtains RMSF from 10 ns simulations of all proteins/peptides. Consequently, the default set of parameters and restraints is devoted to the small-timescale dynamics of proteins. Moreover, the CABS-flex study resulted in RMSFs equivalent to the NMR Ensemble RMSFs [43]. Thus, the binding of TMZ with these proteins could influence the mechanisms involved in gliomagenesis.

For the interaction of biological macromolecules with substrates or protein–protein interactions, flexibility is a critical factor [44]. iMODS is a swift and simple server for defining and calculating the flexibility of a protein. It analyses the molecular motion in addition to the structural flexibility via NMA, which is incorporated with the coordinates of the docked complex [45]. The NMA of proteins is based on the assumption that the vibrational normal modes displaying the minimum frequencies designate the maximum movements in a protein, which are functionally significant [46]. The NMA study of the docked complexes demonstrated considerable mobility, hence proving the structural flexibility of the nine proteins. Moreover, our findings show significant deformability in all of the proteins; most of the proteins were observed to have various peaks with a deformability index of approximately 1.0. As mentioned earlier, deformability is a measure of the flexibility of a given protein, whereas the B-factor is associated with the protein’s mobility [47]. The B-factor analysis of CNTN2-TMZ, SERPINI1-TMZ, OPCML-TMZ, LGI1-TMZ and NPTX1-TMZ generated significant hinges and an approximately average RMS, whereas CREG2-TMZ, GDF1-TMZ, LY6H-TMZ and SLIT1-TMZ produced an insignificant hinge and an average RMS in the B-factor.

The eigenvalues generated for the docked proteins are closely linked to the energy necessary to deform the structure. It signifies the motion stiffness of the protein–ligand complex. The lower the eigenvalue, the easier the deformability of the complex [44]. From the MD study of the docked proteins, we observed that all of the complexes had a considerable amount of deformability. In addition, all of the complexes had low eigenvalues, indicating the good stability and flexibility of the molecular motion of the docked protein complexes. The lowest eigen scores were observed for OPCML, GDF1, CNTN2 and SLIT1 among the nine docked complexes, representing easier deformability and motion stiffness of the protein complexes. The variance maps of all nine complexes yielded reasonable results.

The covariance matrices for CNTN2-TMZ, OPCML-TMZ, GDFI1-TMZ, LY6H-TMZ and SLIT1-TMZ complexes showed good correlations with few anticorrelations, while SERPINI1-TMZ showed considerable correlations with some anticorrelations. LGI1-TMZ, NPTX1-TMZ and CREG2-TMZ indicated equal correlations and anticorrelations. The elastic maps of all nine complexes yielded reasonable results.

Based on the plausible interactions of the selected proteins with TMZ, we speculate that they can serve as potential drug candidates and targets for TMZ to mitigate the pathological consequences associated with GBM pathology.

## 4. Materials and Methods

### 4.1. Retrieval of Ligands and Protein Structures

The 3D structure of Temozolomide (CID:5394) was downloaded in SDF format from a compound database, PubChem (https://pubchem.ncbi.nlm.nih.gov/compound/5394) (accessed on 17 May 2022) [48], and converted into MOL2 format using Open Babel [49]. The selection criteria for protein structures were based on retrieving the FASTA sequence of each protein from UniProt (https://www.uniprot.org/) (accessed on 17 May 2022 [50], followed by PDB advanced BLAST analysis. Structures showing the maximum score and maximum percent identity in BLAST were selected for each protein. The 3D structures of the proteins with more than 97% identity for contactin 2 (PDB ID: 2OM5), SERPINI 1(PDB ID: 3F5N), OPCML (PDB ID: 5UV6), LGI1 (PDB ID: 5Y2Z) and NPTX1 (PDB ID: 6YPE) were directly downloaded from the RCSB Protein Data Bank (PDB) (https://www.rcsb.org/) (accessed on 17 May 2022 [51]. Proteins downloaded from the PDB were then cleaned by deleting duplicated structures and nonstandard residues using BIOVIA Discovery Studio Visualizer [52].

However, proteins having no PDB structures and proteins with less than 97% identity in the PDB database were modeled using Phyre2, an online modeling server (http://www.sbg.bio.ic.ac.uk/phyre2/html/page.cgi?id=index) (accessed on 20 May 2022) [53]. The FASTA sequences of these proteins were submitted to the Phyre2 server, and the top structure with maximum confidence was selected and downloaded for each protein. The modeled protein structures are based on templates for CREG2 (d1xhna1), GDF1 (c3ejrD), LY6H (c2mupA) and SLIT1 (c7cynB).

### 4.2. Pocket Identification

To identify the potential pockets within the structure of each protein, the PDB structures of target proteins were submitted to the DoGSiteScorer server (https://proteins.plus/) (accessed on 20 May 2022) [54]. The selection of the pocket was based on the best drug score. The grid coordinates of the best binding pocket were noted for each protein.

### 4.3. Molecular Docking Analysis

The docking analysis of TMZ with the target proteins was performed using AutoDock 4.2 (https://autodock.scripps.edu/) (accessed on 15 June 2022) [55]. Polar hydrogen and Kollman charges were added to proteins and ligands. Ligand–protein binding energies were measured by setting the grid box based on the predicted coordinates of the best drug score. For each ligand and protein interaction, 20 runs were generated. A complex of all 20 possible ligand–protein poses was established by running Cygwin commands. The best pose based on the lowest binding energy was selected and exported from the complex using PyMol (https://pymol.org/2/) (accessed on 16 June 2022) [56]. Finally, the bonds and interactions between the ligands and the proteins were identified and visualized by BIOVIA Discovery Studio Visualizer.

### 4.4. Molecular Dynamics Simulation Analysis

Molecular dynamics (MD) simulations of protein–ligand complexes were carried out by utilizing CABS-flex V 2.0 (http://biocomp.chem.uw.edu.pl/CABSflex2) (accessed on 20 July 2022) [57] and the iMOD server (iMODS) (http://imods.chaco nlab.org) [46]. CABS-flex was utilized for the assessment of the structural flexibility (RMSF) of all proteins. The simulation time was adjusted to 10 ns, whereas the rest of the parameters were set to default values. The root-mean-square fluctuations (RMSFs) were acquired on the basis of the MD trajectory or NMR ensemble with the default options. To calculate the stability and molecular motion of the docked protein–TMZ complexes, molecular dynamics simulations were performed using the iMOD server. iMODS was utilized for the analysis of the structural dynamics of the docking complexes, along with the determination of the molecular motion. The stability of the protein–TMZ complexes was depicted with reference to its deformability, B-factor, eigenvalues, variance, covariance map and elastic network. The input files were docked PDB files, which were uploaded to the iMODS server, with all parameters set to default.

## 5. Conclusions

Molecular docking and dynamics simulation studies provide a better understanding of the intermolecular-level interactions of TMZ with the circulating/secretory proteins involved in GBM pathogenesis. The present findings provide substantial evidence that these proteins are potential targets of TMZ, highlighting a novel aspect of TMZ’s therapeutic potential. However, extensive in vivo and in vitro studies are warranted to decipher the exact molecular mechanism and mode of action of TMZ by targeting these potential secretory proteins involved in various cellular and molecular pathways associated with GBM.

## Figures and Tables

**Table 1:** Docking interaction of TMZ with the target proteins.

Protein	Ligand	Binding Energy (Kcal/mol)	Conventional H-Bonds	Bond Length (Å)	Other Interaction Types and Interacting Residues	Bond Length (Å)
SLIT1	TMZ	−9.95			Pi-Sigma: VAL 745 Alkyl: ALA 739 Pi-Alkyl: VAL 763 VAL 763 VAL 745 Pi-Anion: ASP 742	2.52 4.73 3.56 4.20 4.34 4.50
GDF1	TMZ	−9.87	LEU147 THR74	2.72 1.93	Pi-Sigma: VAL 194 Pi-Alkyl: PRO 192 LEU 149 LEU147	3.62 4.50 4.71 5.44
NPTX1	TMZ	−8.92	ASN380 LEU394	2.83 3.34	Alkyl: ALA 427 CYS 397 Pi-Alkyl: ALA 401 LEU 394 ALA 427 Pi-Sulfur: CYS 397 Pi-Pi Stacked: HIS 378	3.90 3.25 4.83 4.98 5.19 5.19 4.26
CREG2	TMZ	−8.73			Alkyl: LEU 130 LEU 195 CYS 215 VAL 216 Pi-Alkyl: LEU 130 CYS 215 VAL 216 Pi-Sigma: CYS 215 LEU 130 Pi-Lone Pair: ARG 214	4.05 4.83 4.18 5.08 4.86 3.88 5.04 3.82 3.44 2.86
SERPINI 1	TMZ	−8.06	LEU257 ILE238	2.14 2.07	Pi-Alkyl: ARG259	4.86
OPCML	TMZ	−7.81	ASP61 PRO95	1.93 2.12	C-H Bonds: GLN 97 ASN 93 Alkyl: ILE 59 VAL 66 Pi-Alkyl: PRO 95 VAL 63 VAL 66 TYR 98	3.54 2.95 5.40 3.83 4.89 4.98 4.88 4.37
LGI 1	TMZ	−7.26	SER463 SER463	2.22 2.33	Alkyl: PRO 427 Pi-Alkyl: ILE 426 PRO 427 Pi-Sigma: SER 464 Pi-Sulfur: MET 458	4.39 5.47 3.50 3.67 5.97
CNTN2	TMZ	−6.92	TYR 363 ARG 332 ASP 359	2.20 2.84 1.98	Pi sigma: ARG 342 Alkyl: ARG 342	3.54 4.42
LY6H	TMZ	−6.55	ASN36 SER62 SER68	2.09 1.95 2.67	C-H Bonds: SER 61 ASN 36 Pi-Sulfur: ASP 59	2.77 3.72 3.74

**Table 2:** Eigenvalues of the Docked Proteins Calculated by iMODS.

Protein–Ligand Complex	Eigenvalue
SLIT1-TMZ	5.17 × 10^−5^
GDF1-TMZ	2.17 × 10^−5^
NPTX1-TMZ	1.86 × 10^−3^
CREG2-TMZ	5.36 × 10^−4^
SERPIN1-TMZ	2.64 × 10^−4^
OPCML-TMZ	7.28 × 10^−6^
LGI1-TMZ	2.94 × 10^−4^
CNTN2-TMZ	4.20 × 10^−5^
LY6H-TMZ	1.93 × 10^−4^

## Data Availability

All data generated or analyzed during this study are included in this published article.

## Supplementary Materials

The following supporting information can be downloaded at: https://www.mdpi.com/article/10.3390/molecules27217198/s1. Figures S1–S7: The deformability and B-factor of NPTX1-TMZ, CREG2-TMZ, SERPINI1-TMZ, OPCML-TMZ, LGI1-TMZ, CNTN2-TMZ and LY6H-TMZ; covariance matrices and elastic maps of NPTX1-TMZ, CREG2-TMZ, SERPINI1-TMZ, OPCML-TMZ, LGI1-TMZ, CNTN2-TMZ and LY6H-TMZ. Tables S1–S9, representing the relative propensities of protein residues to diverge from the average dynamic structure.
