# Supplementary material for: Molecular Docking and Molecular Dynamics Studies Reveal Secretory Proteins as Novel Targets of Temozolomide in Glioblastoma Multiforme
Source: Molecules. 2022 Oct 24;27(21):7198. doi: 10.3390/molecules27217198 (PMC9653723; doi:10.3390/molecules27217198)
Supplement: Supplementary file 1 [file molecules-27-07198-s001.zip › molecules-1914169-supplementary.pdf]

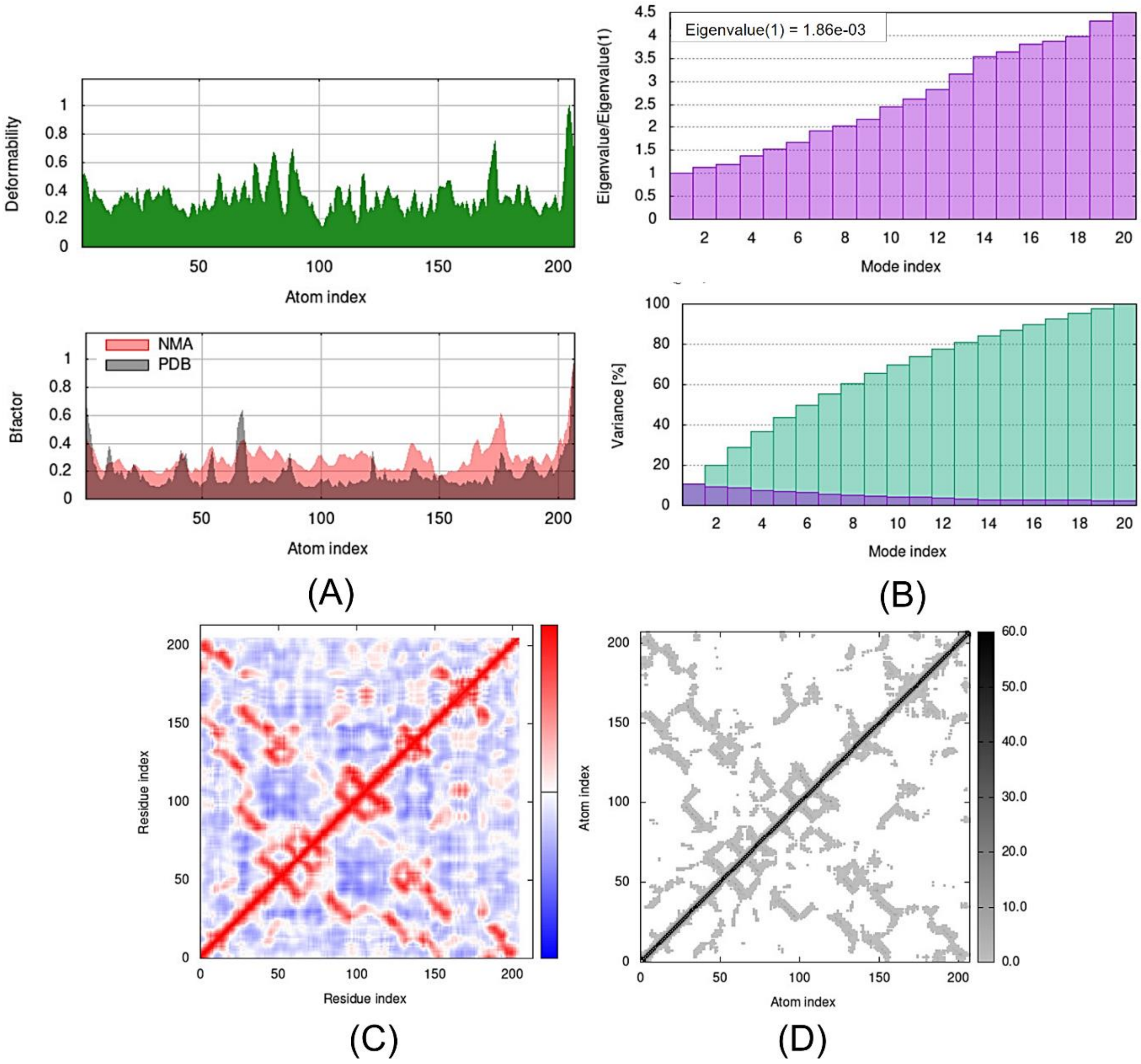

Figure S1. Eigenvalue and variance graphs of NPTX1-TMZ.

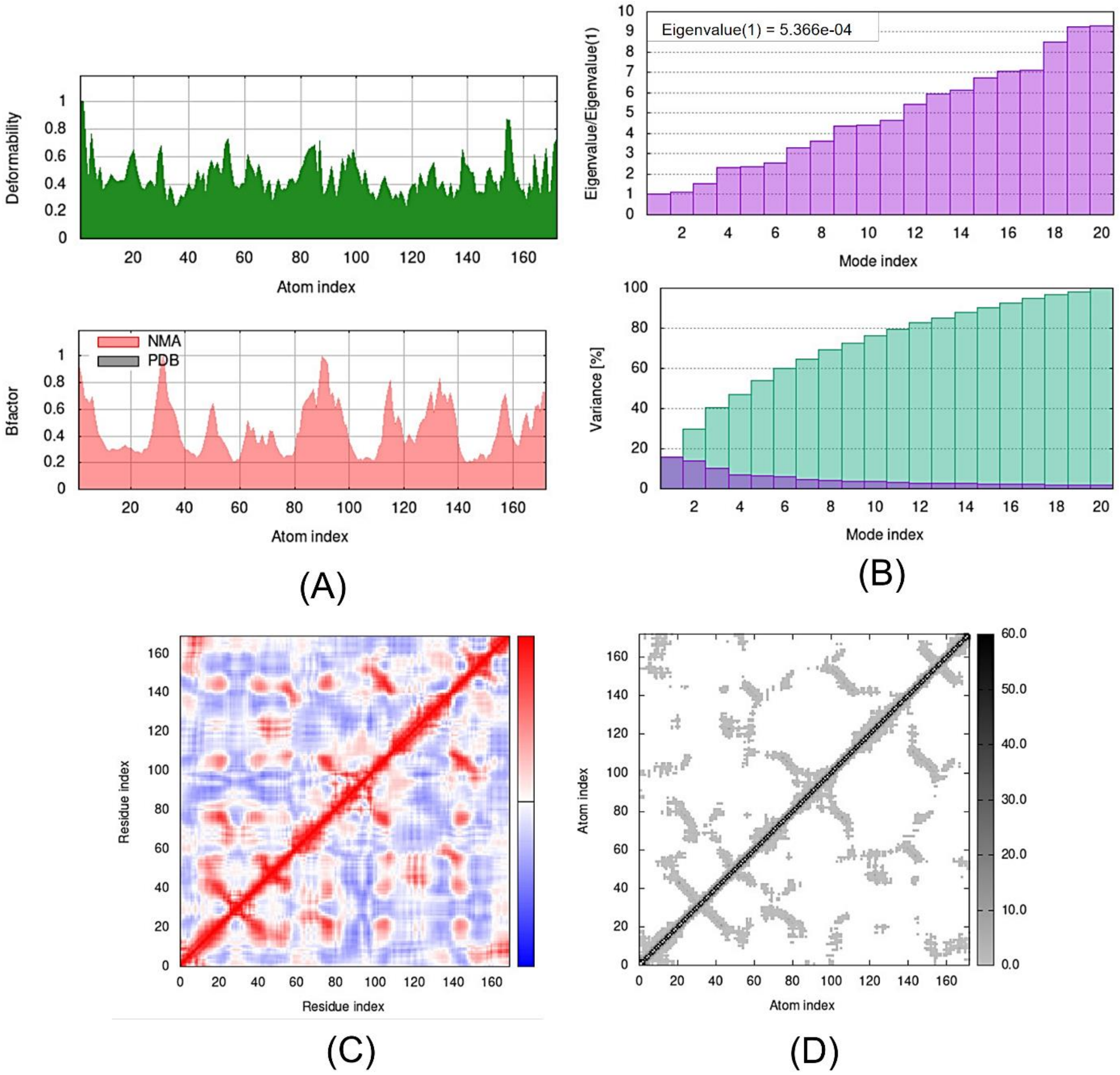

Figure S2. Eigenvalue and variance graphs of CREG2-TMZ.

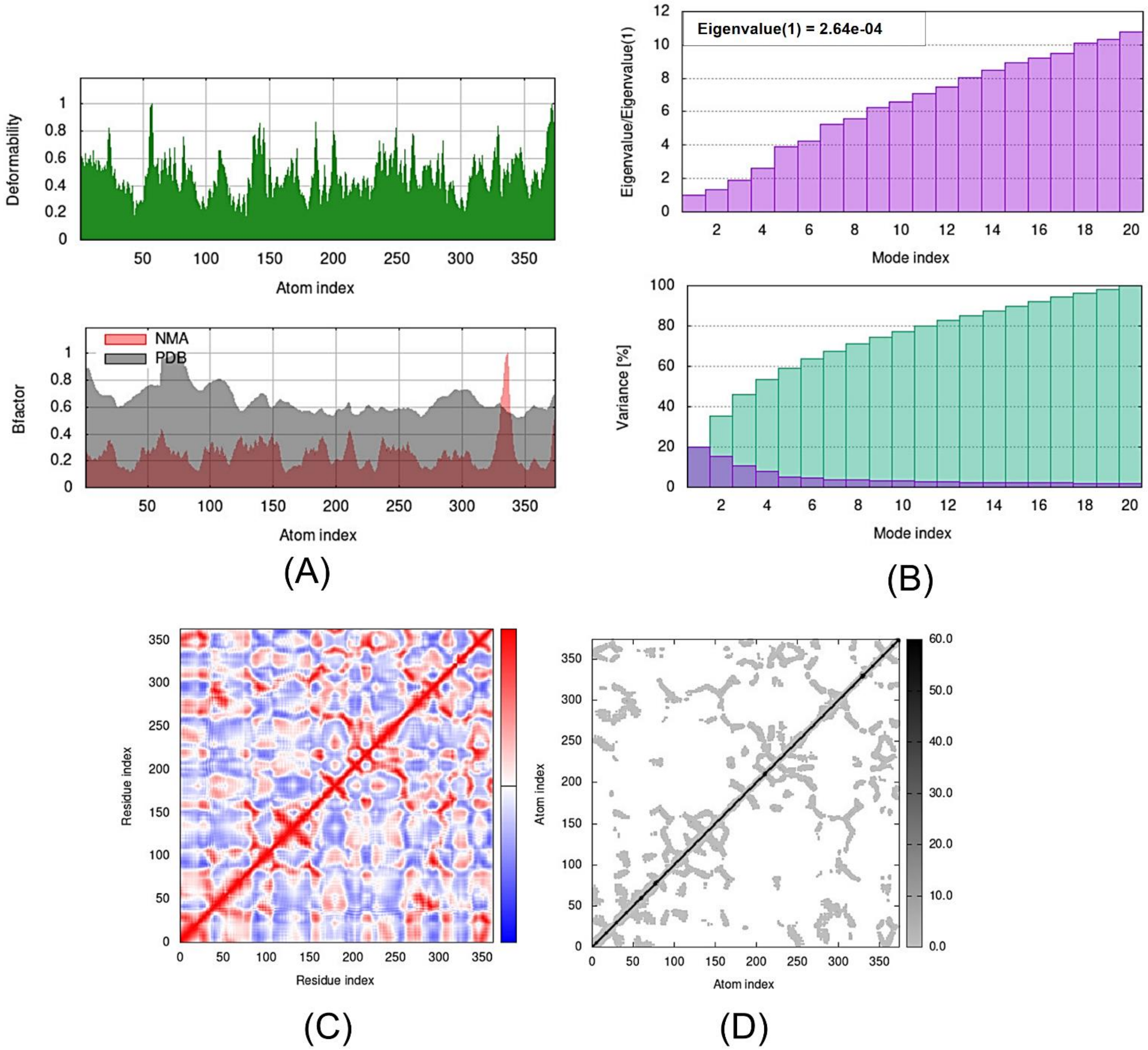

Figure S3. Eigenvalue and variance graphs of SERPINI1-TMZ.

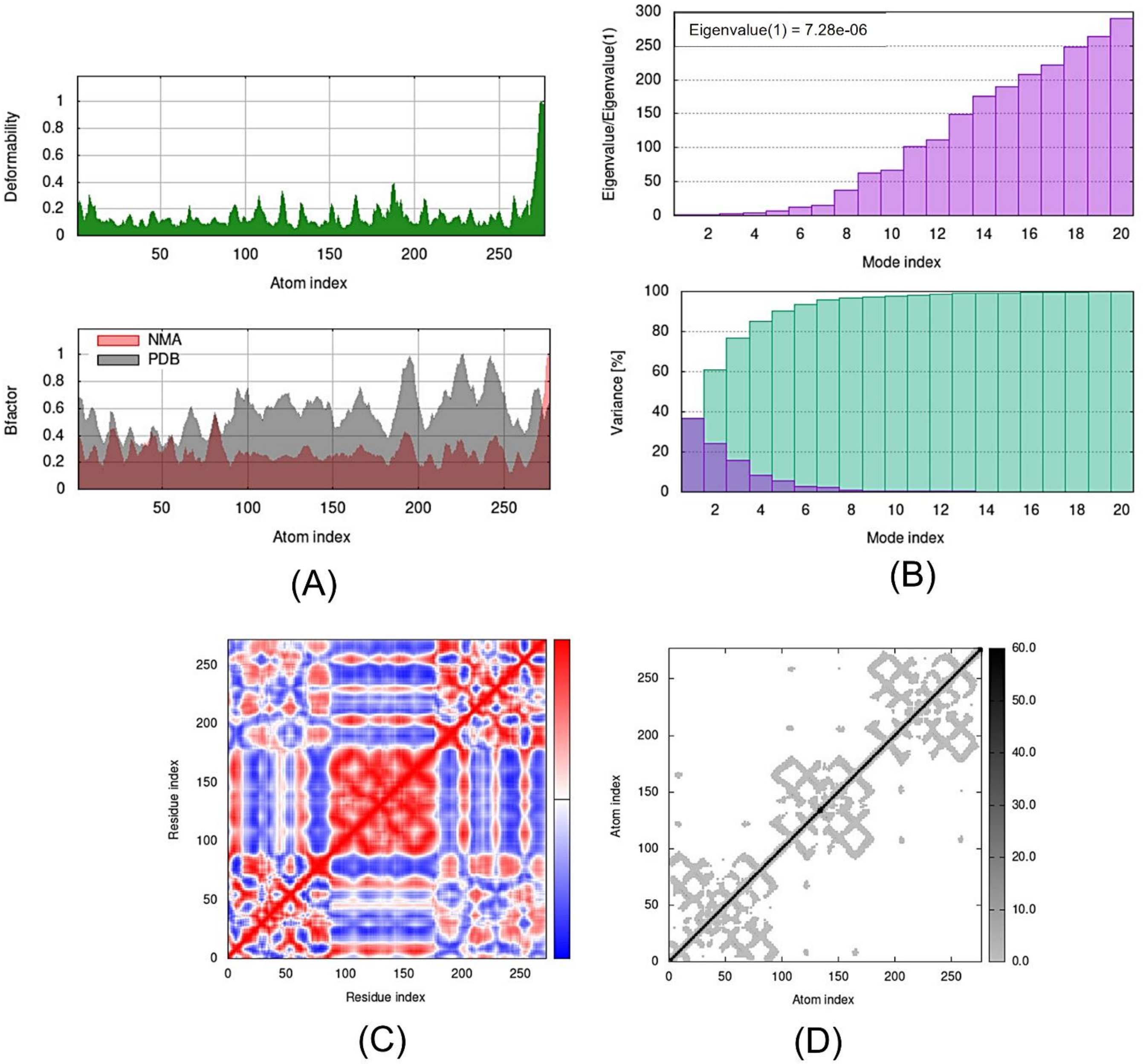

Figure S4. Eigenvalue and variance graphs of OPCML-TMZ.

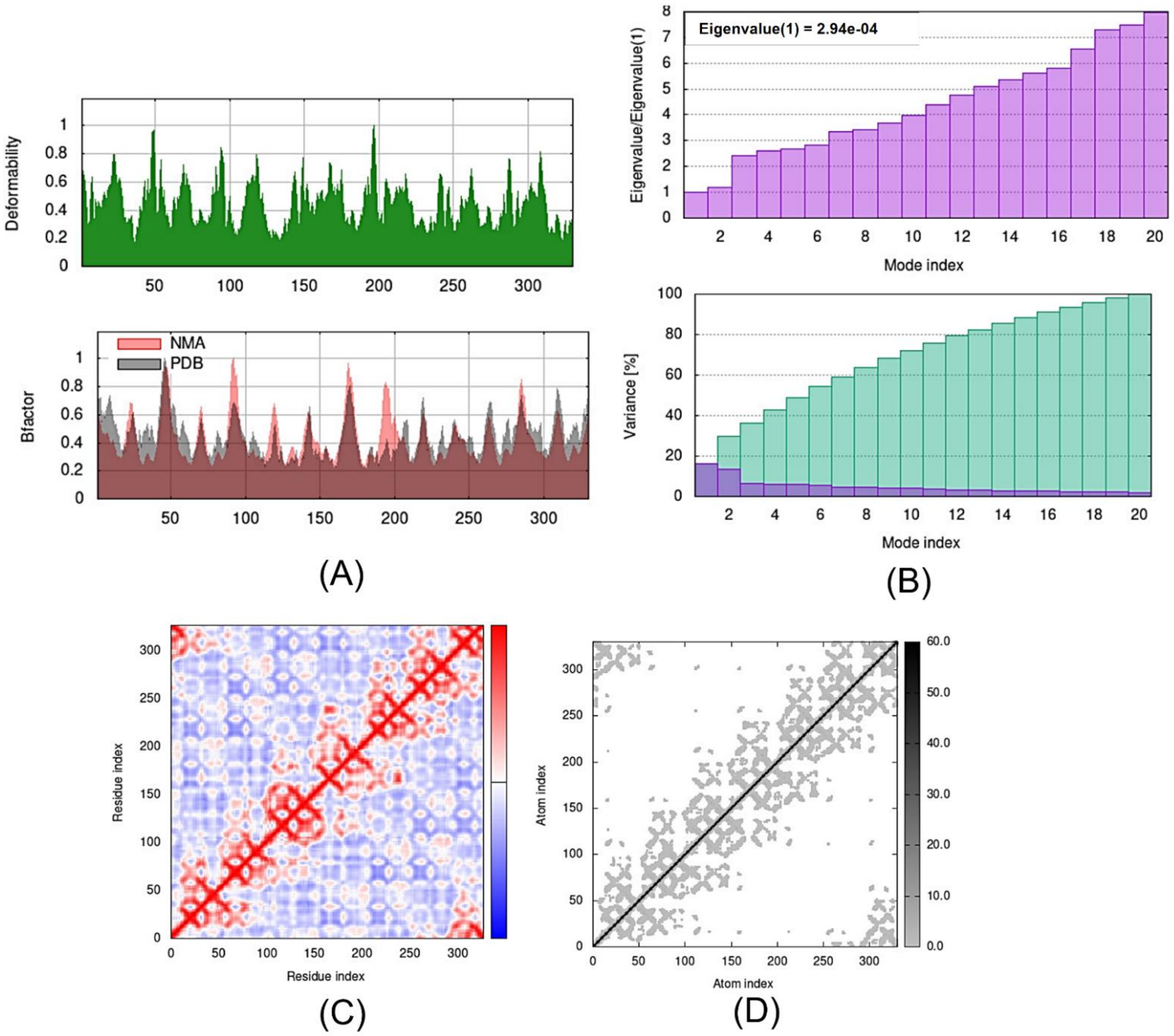

Figure S5. Eigenvalue and variance graphs of LGI1-TMZ.

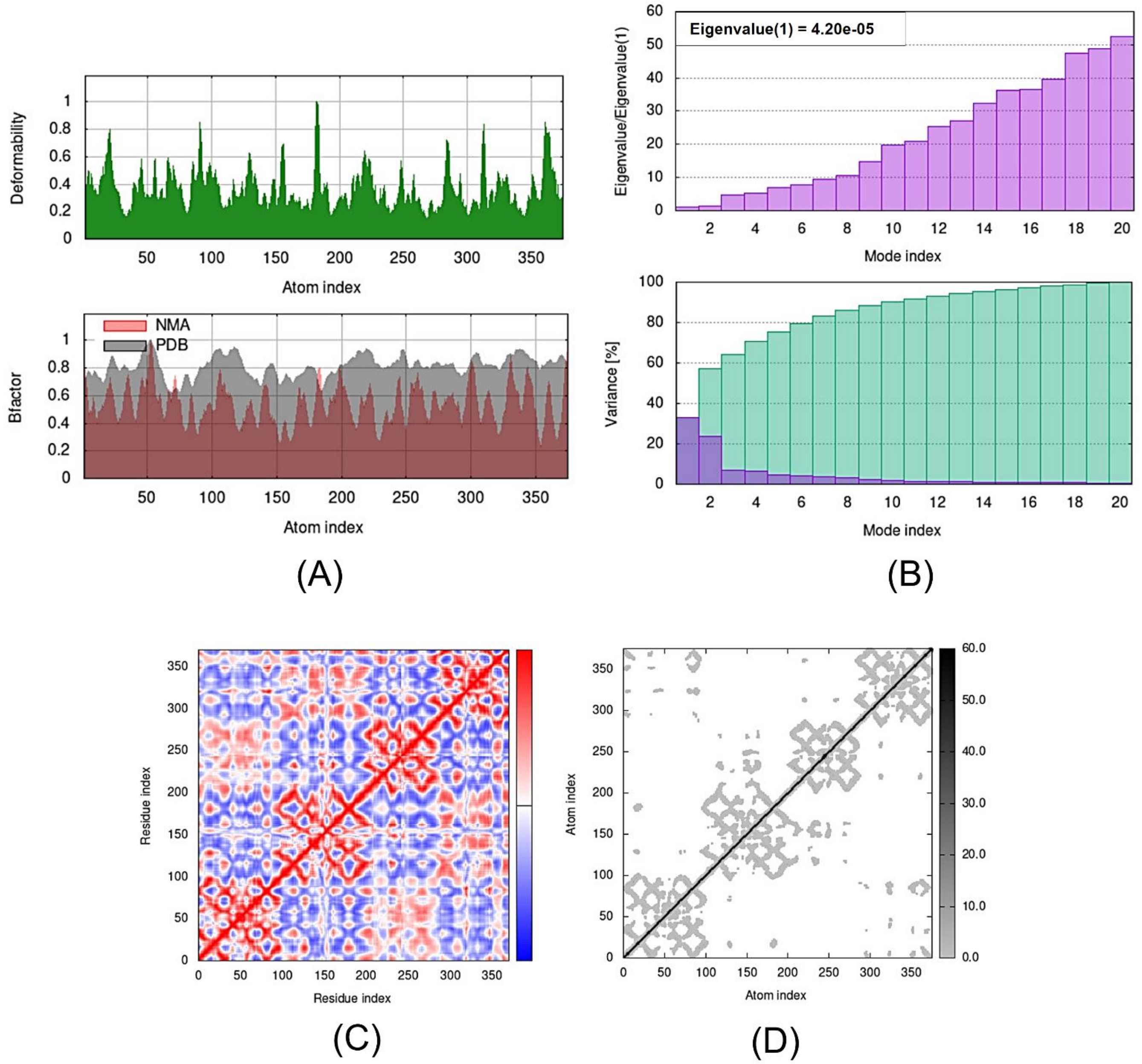

Figure S6. Eigenvalue and variance graphs of CNTN2-TMZ.

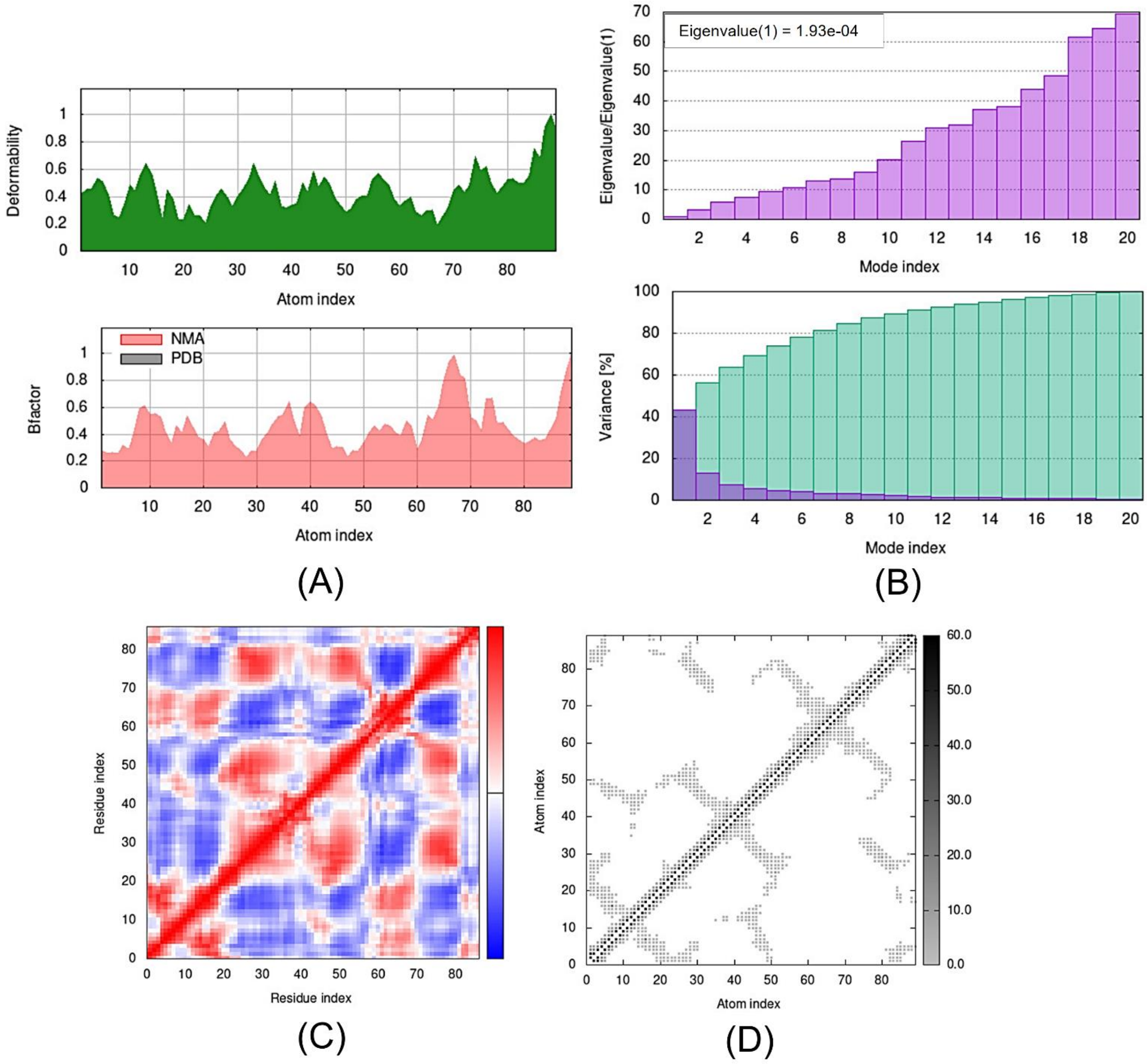

Figure S7. Eigenvalue and variance graphs of LY6H-TMZ.

Supplementary Table S1: RMSF Values Profile of SLIT1

Residue No.	*Chain*	*RMSF Value*
35	A	4.801
36	A	3.645
37	A	3.922
38	A	3.49
39	A	2.512
40	A	2.613
41	A	1.818

42	A	1.609
43	A	1.494
44	A	1.118
45	A	0.846
46	A	0.742
47	A	0.766
48	A	1.594
49	A	1.907
50	A	1.648
51	A	1.988
52	A	1.897
53	A	1.843
54	A	2.452
55	A	2.039
56	A	1.972
57	A	2.437
58	A	1.66
59	A	1.504
60	A	1.608
61	A	2.024
62	A	2.001
63	A	2.004
64	A	0.717
65	A	0.45
66	A	0.321
67	A	0.456
68	A	0.405
69	A	0.665
70	A	0.491
71	A	0.374
72	A	0.132
73	A	0.402
74	A	0.924
75	A	1.842
76	A	2.033
77	A	3.179
78	A	3.515
79	A	2.799
80	A	2.351
81	A	1.371
82	A	1.602
83	A	1.195
84	A	1.332
85	A	1.808
86	A	0.977
87	A	0.994

88	A	0.457
89	A	0.383
90	A	0.303
91	A	0.408
92	A	0.245
93	A	0.284
94	A	0.198
95	A	0.213
96	A	0.209
97	A	0.689
98	A	1.483
99	A	1.481
100	A	1.714
101	A	1.452
102	A	1.453
103	A	1.674
104	A	1.377
105	A	0.904
106	A	0.934
107	A	1.064
108	A	1.113
109	A	0.895
110	A	0.905
111	A	1.39
112	A	1.594
113	A	1.49
114	A	1.647
115	A	1.327
116	A	1.32
117	A	1.188
118	A	1.033
119	A	1.394
120	A	2.282
121	A	1.911
122	A	1.756
123	A	0.943
124	A	0.92
125	A	0.947
126	A	1.089
127	A	1.529
128	A	1.668
129	A	1.158
130	A	2.574
131	A	2.561
132	A	2.742
133	A	1.431

134	A	1.401
135	A	0.323
136	A	0.187
137	A	0.077
138	A	0.166
139	A	0.185
140	A	0.256
141	A	0.359
142	A	0.361
143	A	0.681
144	A	0.959
145	A	2.093
146	A	1.819
147	A	2.616
148	A	1.138
149	A	1.675
150	A	0.901
151	A	0.731
152	A	0.582
153	A	0.594
154	A	0.601
155	A	1.724
156	A	1.809
157	A	1.115
158	A	0.933
159	A	0.239
160	A	0.127
161	A	0.075
162	A	0.099
163	A	0.095
164	A	0.573
165	A	0.496
166	A	0.695
167	A	0.793
168	A	1.337
169	A	1.098
170	A	1.767
171	A	1.218
172	A	1.141
173	A	1.485
174	A	2.005
175	A	3.336
176	A	3.619
177	A	1.656
178	A	1.848
179	A	0.892

180	A	0.618
181	A	0.635
182	A	0.505
183	A	0.353
184	A	0.398
185	A	0.168
186	A	0.091
187	A	0.089
188	A	0.232
189	A	0.291
190	A	0.271
191	A	0.711
192	A	2.18
193	A	2.24
194	A	3.107
195	A	4.658
196	A	4.603
197	A	4.546
198	A	2.843
199	A	2.465
200	A	1.842
201	A	2.209
202	A	1.018
203	A	1.087
204	A	0.882
205	A	1.313
206	A	2.018
207	A	1.943
208	A	2.184
209	A	1.154
210	A	0.948
211	A	0.993
212	A	0.869
213	A	0.781
214	A	0.986
215	A	0.878
291	A	0.428
292	A	0.614
293	A	0.409
294	A	0.381
295	A	0.391
296	A	0.598
297	A	0.707
298	A	0.71
299	A	0.95
300	A	1.102

301	A	1.682
302	A	2.497
303	A	1.104
304	A	1.745
305	A	1.183
306	A	0.911
307	A	1.078
308	A	1.255
309	A	1.276
310	A	0.736
311	A	0.536
312	A	0.557
313	A	0.427
314	A	0.274
315	A	0.67
316	A	0.427
317	A	0.489
318	A	0.842
319	A	0.841
320	A	1.155
321	A	1.094
322	A	1.368
323	A	1.201
324	A	1.03
325	A	1.201
326	A	1.255
327	A	0.866
328	A	0.72
329	A	0.505
330	A	0.669
331	A	0.655
332	A	0.485
333	A	0.756
334	A	0.667
335	A	0.548
336	A	0.418
337	A	0.511
338	A	0.335
339	A	0.606
340	A	0.552
341	A	1.312
342	A	0.543
343	A	0.661
344	A	1.145
345	A	1.302
346	A	1.671

347	A	1.015
348	A	1.698
349	A	2.169
350	A	3.685
351	A	4.561
352	A	3.903
353	A	2.744
354	A	3.164
355	A	3.32
356	A	2.827
357	A	1.928
358	A	2.746
359	A	2.045
360	A	1.733
361	A	1.458
362	A	1.155
363	A	0.829
364	A	0.988
365	A	0.935
366	A	1.201
367	A	1.424
399	A	2.514
400	A	1.71
401	A	1.345
402	A	2.045
403	A	1.65
404	A	0.699
405	A	1.524
406	A	0.418
407	A	0.354
408	A	0.181
409	A	0.093
410	A	0.323
411	A	0.656
412	A	0.704
413	A	0.766
414	A	0.756
415	A	1.409
416	A	1.542
417	A	2.395
418	A	1.887
419	A	1.677
420	A	1.53
421	A	1.46
422	A	1.218
423	A	1.41

424	A	1.111
425	A	0.83
426	A	1.36
427	A	2.485
428	A	2.064
429	A	1.952
430	A	0.987
431	A	0.451
432	A	0.499
433	A	0.221
434	A	0.099
435	A	0.24
436	A	0.491
437	A	0.617
438	A	0.69
439	A	0.705
440	A	0.877
441	A	1.673
442	A	1.181
443	A	0.932
444	A	0.63
445	A	0.387
446	A	0.303
447	A	0.361
448	A	0.382
449	A	0.126
450	A	0.417
451	A	0.575
452	A	0.457
453	A	0.635
454	A	0.903
455	A	0.649
456	A	2.203
457	A	1.821
458	A	0.958
459	A	1.411
460	A	1.836
461	A	1.874
462	A	1.989
463	A	2.65
464	A	1.907
465	A	0.822
466	A	0.809
467	A	0.322
468	A	0.371
469	A	0.124

470	A	0.05
471	A	0.051
472	A	0.055
473	A	0.694
474	A	1.341
475	A	1.795
476	A	2.246
477	A	1.629
478	A	1.046
479	A	0.882
480	A	0.899
481	A	1.015
482	A	1.192
483	A	1.143
484	A	1.538
485	A	1.103
486	A	1.212
487	A	1.221
488	A	0.976
489	A	0.966
490	A	1.492
491	A	1.298
492	A	2.3
493	A	1.602
494	A	1.407
495	A	0.652
496	A	0.541
497	A	0.461
498	A	0.345
499	A	0.098
500	A	0.048
501	A	0.05
502	A	0.052
503	A	0.53
504	A	0.575
505	A	0.674
506	A	0.723
507	A	0.802
508	A	0.846
509	A	0.92
510	A	1.184
511	A	0.877
512	A	0.603
513	A	0.932
514	A	0.874
515	A	0.741

516	A	1.389
517	A	1.194
518	A	1.428
519	A	1.688
520	A	1.609
521	A	1.057
522	A	0.946
523	A	0.318
524	A	0.057
525	A	0.05
526	A	0.354
527	A	1.212
528	A	1.098
529	A	1.9
530	A	2.505
531	A	2.249
532	A	1.663
533	A	1.125
534	A	1.169
557	A	1.23
558	A	1.408
559	A	1.643
560	A	1.393
561	A	1.342
562	A	1.853
563	A	1.956
564	A	1.242
565	A	1.7
566	A	1.202
567	A	0.638
568	A	0.386
569	A	0.059
570	A	0.095
571	A	0.046
572	A	0.218
573	A	0.045
574	A	0.046
575	A	0.047
576	A	0.046
577	A	0.044
578	A	0.534
579	A	0.94
580	A	1.717
581	A	2.008
582	A	2.101
583	A	1.555

584	A	0.992
585	A	0.992
586	A	2.674
587	A	2.575
588	A	2.336
589	A	2.833
590	A	3.106
591	A	1.353
592	A	0.92
593	A	0.263
594	A	0.049
595	A	0.045
596	A	0.046
597	A	0.045
598	A	0.046
599	A	0.049
600	A	0.052
601	A	0.783
602	A	0.921
603	A	1.255
604	A	1.239
605	A	1.146
606	A	1.855
607	A	3.585
608	A	2.896
609	A	1.338
610	A	2.005
611	A	1.425
612	A	0.948
613	A	0.809
614	A	0.79
615	A	0.69
616	A	0.286
617	A	0.185
618	A	0.049
619	A	0.047
620	A	0.048
621	A	0.062
622	A	0.093
623	A	0.53
624	A	0.809
625	A	0.638
626	A	0.819
627	A	0.668
628	A	0.387
629	A	0.732

630	A	0.854
631	A	0.83
632	A	1.003
633	A	1.514
634	A	1.778
635	A	1.342
636	A	1.135
637	A	0.908
638	A	0.533
639	A	0.265
640	A	0.173
641	A	0.056
642	A	0.05
643	A	0.048
644	A	0.049
645	A	0.176
646	A	0.227
647	A	0.354
648	A	0.339
649	A	0.269
650	A	0.451
651	A	0.546
652	A	0.698
653	A	1.267
654	A	1.333
655	A	1.69
656	A	2.821
657	A	1.694
658	A	1.773
659	A	0.983
660	A	0.795
661	A	0.576
662	A	0.375
663	A	0.267
664	A	0.061
665	A	0.054
666	A	0.053
667	A	0.05
668	A	0.052
669	A	0.181
670	A	0.235
671	A	0.341
672	A	0.53
673	A	0.594
674	A	0.899
675	A	0.97

676	A	0.976
677	A	0.565
678	A	0.361
679	A	0.387
680	A	0.369
681	A	0.547
682	A	0.452
683	A	0.539
684	A	0.162
685	A	0.198
686	A	0.238
687	A	0.23
688	A	0.127
689	A	0.414
690	A	0.561
691	A	0.422
692	A	0.055
693	A	0.052
694	A	0.054
695	A	0.561
696	A	0.823
697	A	0.716
698	A	0.489
699	A	0.833
700	A	0.567
701	A	0.669
702	A	0.637
703	A	0.657
704	A	0.824
705	A	0.699
706	A	0.883
707	A	0.577
708	A	0.822
709	A	0.855
710	A	1.44
711	A	0.836
712	A	1.047
713	A	0.851
714	A	0.53
715	A	0.058
716	A	0.055
717	A	0.058
718	A	0.057
719	A	0.582
720	A	0.516
721	A	0.972

722	A	1.92
723	A	1.542
724	A	1.026
725	A	1.73
726	A	2.973
727	A	4.035
728	A	3.714
729	A	3.224
730	A	2.629
731	A	1.247
732	A	0.823
733	A	0.589
734	A	0.735
735	A	0.962
736	A	1.498
737	A	1.138
738	A	1.568
739	A	0.673
740	A	1.702
741	A	1.182
742	A	2.217
743	A	0.784
744	A	0.587
745	A	0.061
746	A	0.058
747	A	0.061
748	A	0.06
749	A	0.37
750	A	0.467
751	A	0.679
752	A	0.391
753	A	0.394
754	A	0.413
755	A	0.928
756	A	0.746
757	A	0.579
758	A	0.65
759	A	0.576
760	A	0.765
761	A	1.079
762	A	1.139
763	A	0.965
764	A	0.477
765	A	0.067
766	A	0.065
767	A	0.064

768	A	0.077
769	A	0.089
770	A	0.561
771	A	0.887
772	A	0.519
773	A	0.574
774	A	0.618
775	A	0.823
776	A	1.079
777	A	1.192
778	A	1.395
779	A	1.373
780	A	0.964
781	A	1.191
782	A	1.195
783	A	0.898
784	A	1.17
785	A	0.967
786	A	0.657
787	A	0.283
788	A	0.236
789	A	0.315
790	A	0.596
791	A	0.393
792	A	0.944
793	A	1.836
794	A	1.867
795	A	1.69
796	A	1.114
797	A	1.853
798	A	1.425
799	A	1.515
800	A	1.175
801	A	1.641
802	A	1.434
803	A	1.014
804	A	1.126
805	A	1.342
806	A	1.097
807	A	0.858
808	A	1.006
809	A	0.585
810	A	0.537
811	A	0.686
812	A	0.28
813	A	0.32

814	A	0.49
815	A	0.322
816	A	0.257
817	A	0.64
818	A	0.746
819	A	0.921
820	A	0.838
821	A	1.189
822	A	0.993
823	A	1.328
824	A	1.727
825	A	1.931
826	A	1.439
827	A	1.163
828	A	0.861
829	A	1.025
830	A	1.349
831	A	1.333
832	A	1.25
833	A	0.773
834	A	0.506
835	A	0.509
836	A	0.088
837	A	0.108
838	A	0.135
839	A	0.138
840	A	0.343
841	A	0.501
842	A	0.531
843	A	0.778
844	A	0.75
845	A	1.044
846	A	1.194
847	A	0.647
848	A	1.047
849	A	1.674
850	A	0.932
851	A	0.474
852	A	0.33
853	A	0.602
854	A	0.927
855	A	0.914
856	A	0.964
857	A	1.611
858	A	0.799
859	A	0.982

860	A	0.688
861	A	0.304
862	A	0.471
863	A	0.425
864	A	0.646
865	A	0.843
866	A	0.737
867	A	0.733
868	A	0.479
869	A	0.672
870	A	0.762
871	A	1.443
872	A	1.137
873	A	0.822
874	A	0.887
875	A	0.979
876	A	1.11
877	A	0.745
878	A	0.516
879	A	1.021
880	A	1.044
881	A	0.541
882	A	0.787
883	A	1.195
884	A	1.407
885	A	1.089
886	A	1.358
887	A	1.519
888	A	2.003
889	A	1.137
890	A	0.578
891	A	0.715
892	A	0.651
893	A	0.566
894	A	0.726
895	A	1.157
896	A	2.44
897	A	2.722
898	A	2.997
899	A	2.534
900	A	2.446
901	A	1.535
902	A	0.776
903	A	0.394
904	A	0.556
905	A	0.641

906	A	0.809
907	A	0.772
908	A	1.175
909	A	1.767
910	A	2.139
911	A	3.146
912	A	4.03
913	A	4.324
914	A	5.104
915	A	5.796
916	A	7.178
917	A	8.078

Supplementary Table S2: RMSF Values Profile of GDF1

Residue No.	*Chain*	*RMSF Value*
33	A	0.964
34	A	0.565
35	A	0.596
36	A	0.597
37	A	0.452
38	A	0.509
39	A	0.526
40	A	0.397
41	A	0.352
42	A	0.434
43	A	0.57
44	A	1.013
45	A	2.053
46	A	2.326
47	A	3.113
48	A	3.121
49	A	3.169
50	A	3.152
51	A	2.807
52	A	2.703
53	A	2.26
54	A	3.261
55	A	2.683
56	A	2.411
57	A	2.853
58	A	2.443
59	A	2.25
60	A	2.096

61	A	1.803
62	A	1.653
63	A	1.645
64	A	1.639
65	A	1.29
66	A	1.312
67	A	1.565
68	A	1.462
69	A	1.312
70	A	1.287
71	A	1.379
72	A	1.276
73	A	0.976
74	A	1.055
75	A	1.411
76	A	2.335
77	A	6.242
78	A	5.72
79	A	4.977
80	A	4.275
81	A	3.858
82	A	2.223
83	A	1.913
84	A	1.337
85	A	0.958
99	A	0.986
100	A	0.981
101	A	0.758
102	A	0.587
103	A	0.436
104	A	0.506
105	A	0.497
106	A	0.734
107	A	2.189
108	A	2.096
109	A	3.205
110	A	3.489
111	A	2.887
112	A	1.547
113	A	1.461
114	A	2.278
115	A	2.732
116	A	3.113
117	A	3.016
118	A	2.31
119	A	3.087

120	A	3.452
121	A	2.579
122	A	2.242
123	A	1.771
124	A	1.381
125	A	1.177
126	A	0.731
127	A	0.452
128	A	0.3
129	A	0.362
130	A	0.343
131	A	0.296
132	A	0.743
133	A	0.97
134	A	1.528
135	A	1.23
136	A	0.768
137	A	1.227
138	A	1.629
139	A	2.235
140	A	2.874
141	A	2.003
142	A	1.615
143	A	0.888
144	A	0.542
145	A	0.395
146	A	0.109
147	A	0.451
148	A	0.211
149	A	0.197
150	A	0.107
151	A	0.118
152	A	0.362
153	A	0.855
154	A	1.609
155	A	2.163
156	A	2.056
157	A	1.562
158	A	2.076
159	A	2.252
160	A	2.156
161	A	1.731
162	A	0.834
163	A	0.39
164	A	0.315
165	A	0.276

166	A	0.159
167	A	0.307
168	A	0.554
169	A	0.307
170	A	0.634
171	A	1.819
172	A	1.693
173	A	1.763
174	A	2.354
175	A	1.412
176	A	0.989
177	A	0.721
178	A	0.973
179	A	0.914
180	A	0.9
181	A	0.59
182	A	0.549
183	A	0.404
184	A	0.484
185	A	0.946
186	A	1.383
187	A	1.372
188	A	1.952
189	A	1.732
190	A	0.902
191	A	0.856
192	A	0.589
193	A	0.579
194	A	0.361
195	A	0.613
196	A	0.563
197	A	0.544
198	A	1.112
199	A	0.779
200	A	0.792
201	A	1.064
202	A	0.731
203	A	0.648
204	A	0.832
205	A	1.04
206	A	1.145
207	A	2.175
208	A	2.003
209	A	1.803
210	A	0.886
211	A	0.486

212	A	0.4
213	A	0.113
214	A	0.401
215	A	0.116
216	A	0.232
217	A	0.231
218	A	0.339
219	A	0.909
220	A	2.374
221	A	3.342
222	A	3.696
223	A	4.197
224	A	4.278
225	A	2.766
226	A	1.127
227	A	0.526
228	A	0.626
229	A	1.562
230	A	2.129
231	A	1.753
232	A	2.384
233	A	1.396
234	A	0.878
235	A	0.468
236	A	0.409
237	A	0.117
238	A	0.418
239	A	0.298
240	A	0.567
241	A	0.804
242	A	0.897
243	A	1.067
244	A	1.359
245	A	1.342
246	A	1.986
247	A	2.661
248	A	2.526
249	A	3.193
250	A	2.438
251	A	2.509
252	A	2.816
253	A	1.909
254	A	0.685
255	A	1.12
256	A	1.103
257	A	1.398

258	A	1.333
259	A	1.008
260	A	1.597
261	A	1.638
262	A	1.662
263	A	1.824
264	A	2.354
265	A	1.945
266	A	1.006
267	A	0.717
268	A	0.225
269	A	0.253
270	A	0.652
271	A	0.714
272	A	0.564
273	A	0.635
274	A	0.662
275	A	0.834
276	A	0.612
277	A	0.894
278	A	0.96
279	A	0.758
280	A	0.761
281	A	0.408
282	A	0.805
283	A	0.708
284	A	0.7
285	A	0.585
286	A	0.719
287	A	0.61
288	A	1.274
289	A	1.344
290	A	0.914
291	A	0.508
292	A	0.415
293	A	0.433
294	A	0.334
295	A	0.291
296	A	0.259
297	A	0.45
298	A	1.241
299	A	1.252
300	A	1.076
301	A	1.363
302	A	1.158
303	A	1.674

304	A	1.318
305	A	1.885
306	A	1.294
307	A	1.567
308	A	1.57
309	A	1.235
310	A	1.095
311	A	0.988
312	A	0.829
313	A	0.52
314	A	0.422
315	A	0.492
316	A	0.325
317	A	0.164
318	A	0.16
319	A	0.283
320	A	0.149
321	A	0.137
322	A	0.28
323	A	0.185
324	A	0.221
325	A	0.148
326	A	0.539
327	A	0.547
328	A	0.675
329	A	1.093
330	A	1.73
331	A	1.923
332	A	1.947
333	A	2.117
334	A	1.744
335	A	2.054
336	A	1.138
337	A	0.619
338	A	0.732
339	A	0.618
340	A	0.948
341	A	0.779
342	A	0.333
343	A	0.418
344	A	0.42
345	A	0.313
346	A	0.439
347	A	0.505
348	A	0.499
349	A	0.573

350	A	0.596
351	A	1.018
352	A	2.767
353	A	2.826
354	A	2.566
355	A	1.174
356	A	0.752
357	A	0.728
358	A	0.746
359	A	0.849
360	A	0.712
361	A	0.626
362	A	0.477
363	A	0.624
364	A	0.501
365	A	0.218
366	A	0.343
367	A	0.705
368	A	0.783
369	A	0.298
370	A	0.293
371	A	0.672

Supplementary Table S3: RMSF Values Profile of NPTX1

Residue No.	*Chain*	*RMSF Value*
225	A	3.185
226	A	1.585
227	A	0.856
228	A	0.474
229	A	0.201
230	A	0.24
231	A	0.52
232	A	0.459
233	A	0.53
234	A	0.497
235	A	1.038
236	A	1.166
237	A	1.39
238	A	0.821
239	A	0.266
240	A	0.112
241	A	0.129
242	A	0.315

243	A	0.151
244	A	0.216
245	A	0.246
246	A	0.203
247	A	0.14
248	A	0.161
249	A	0.133
250	A	0.104
251	A	0.121
252	A	0.107
253	A	0.102
254	A	0.092
255	A	0.407
256	A	0.316
257	A	0.126
258	A	0.118
259	A	0.299
260	A	0.351
261	A	0.388
262	A	0.925
263	A	1.465
264	A	1.002
265	A	1.024
266	A	0.589
267	A	0.567
268	A	0.58
269	A	0.252
270	A	0.092
271	A	0.29
272	A	0.087
273	A	0.09
274	A	0.11
275	A	0.294
276	A	0.668
277	A	0.91
278	A	0.629
279	A	0.405
280	A	0.398
281	A	0.107
282	A	0.09
283	A	0.094
284	A	0.319
285	A	0.327
286	A	0.473
287	A	1.366
288	A	1.308

289	A	2.355
290	A	2.553
291	A	1.333
292	A	0.576
293	A	0.372
294	A	0.361
295	A	0.298
296	A	0.624
297	A	0.875
298	A	1.555
299	A	0.765
300	A	0.476
301	A	0.356
302	A	0.563
303	A	0.56
304	A	0.718
305	A	0.458
306	A	1.015
307	A	0.446
308	A	0.502
309	A	0.696
310	A	0.71
311	A	0.275
312	A	0.363
313	A	0.356
314	A	0.401
315	A	0.374
316	A	0.544
317	A	0.399
318	A	0.288
319	A	0.122
320	A	0.159
321	A	0.165
322	A	0.324
323	A	0.518
324	A	0.633
325	A	0.369
326	A	0.31
327	A	0.273
328	A	0.144
329	A	0.39
330	A	0.358
331	A	0.816
332	A	1.577
333	A	1.03
334	A	0.605

335	A	0.709
336	A	0.482
337	A	0.491
338	A	0.688
339	A	0.882
340	A	1.325
341	A	2.312
342	A	1.359
343	A	0.563
344	A	0.421
345	A	0.502
346	A	0.418
347	A	0.107
348	A	0.112
349	A	0.139
350	A	0.147
351	A	0.131
352	A	0.138
353	A	0.099
354	A	0.141
355	A	0.1
356	A	0.311
357	A	0.111
358	A	0.388
359	A	0.63
360	A	0.866
361	A	1.101
362	A	2.706
363	A	3.177
364	A	1.617
365	A	1.302
366	A	0.823
367	A	0.819
368	A	1.311
369	A	1.135
370	A	0.616
371	A	0.69
372	A	0.73
373	A	0.625
374	A	0.492
375	A	0.517
376	A	0.118
377	A	0.138
378	A	0.162
379	A	0.094
380	A	0.275

381	A	0.089
382	A	0.093
383	A	0.131
384	A	0.143
385	A	0.162
386	A	0.165
387	A	0.448
388	A	0.532
389	A	0.523
390	A	0.445
391	A	0.6
392	A	0.387
393	A	0.403
394	A	0.614
395	A	0.766
396	A	0.923
397	A	0.736
398	A	0.962
399	A	1.079
400	A	1.05
401	A	0.972
402	A	1.717
403	A	1.382
404	A	2.417
405	A	1.204
406	A	0.919
407	A	0.461
408	A	0.42
409	A	0.457
410	A	0.463
411	A	0.399
412	A	0.698
413	A	0.485
414	A	0.343
415	A	0.2
416	A	0.19
417	A	0.422
418	A	0.566
419	A	0.893
420	A	0.447
421	A	0.454
422	A	0.725
423	A	0.623
424	A	0.643
425	A	0.965
426	A	0.954

427	A	0.798
428	A	0.623
429	A	1.085

Supplementary Table S4: RMSF Values Profile of CREG2

Residue No.	*Chain*	*RMSF Value*
117	A	2.306
118	A	2.34
119	A	2.607
120	A	1.959
121	A	1.538
122	A	1.005
123	A	0.558
124	A	0.394
125	A	0.395
126	A	0.364
127	A	0.296
128	A	0.332
129	A	0.442
130	A	0.289
131	A	0.359
132	A	0.469
133	A	0.561
134	A	0.448
135	A	0.306
136	A	0.19
137	A	0.159
138	A	0.173
139	A	0.153
140	A	0.336
141	A	0.545
142	A	0.678
143	A	1.776
144	A	3.07
145	A	4.122
146	A	5.481
147	A	5.852
148	A	5.186
149	A	3.373
150	A	2.535
151	A	1.861
152	A	0.879
153	A	0.967
154	A	0.783

155	A	0.427
156	A	0.402
157	A	0.304
158	A	0.302
159	A	0.355
160	A	0.266
161	A	0.581
162	A	1.263
163	A	3.045
164	A	4.813
165	A	4.841
166	A	4.016
167	A	2.901
168	A	1.827
169	A	1.022
170	A	0.597
171	A	0.563
172	A	0.181
173	A	0.221
174	A	0.274
175	A	0.369
176	A	0.626
177	A	2.365
178	A	2.214
179	A	2.212
180	A	1.323
181	A	1.029
182	A	1.114
183	A	0.902
184	A	0.543
185	A	0.884
186	A	1.147
187	A	0.766
188	A	0.749
189	A	0.641
190	A	0.64
191	A	0.413
192	A	0.295
193	A	0.121
194	A	0.178
195	A	0.172
196	A	0.322
197	A	0.483
198	A	0.561
199	A	0.691
200	A	1.128

201	A	0.967
202	A	0.714
203	A	0.59
204	A	0.816
205	A	0.736
206	A	0.706
207	A	0.954
208	A	1.091
209	A	1.191
210	A	1.456
211	A	1.391
212	A	1.293
213	A	0.589
214	A	0.524
215	A	0.346
216	A	0.408
217	A	0.166
218	A	0.14
219	A	0.151
220	A	0.117
221	A	0.157
222	A	0.432
223	A	0.471
224	A	0.49
225	A	0.776
226	A	0.879
227	A	1.415
228	A	2.833
229	A	2.706
230	A	2.943
231	A	2.132
232	A	1.921
233	A	1.924
234	A	1.715
235	A	1.016
236	A	1.256
237	A	1.423
238	A	1.023
239	A	1.142
240	A	1.448
241	A	1.596
242	A	0.952
243	A	1.451
244	A	2.001
245	A	2.778
246	A	3.499

247	A	1.748
248	A	1.891
249	A	1.68
250	A	1.931
251	A	1.738
252	A	1.999
253	A	1.786
254	A	1.709
255	A	0.617
256	A	0.487
257	A	0.303
258	A	0.216
259	A	0.413
260	A	0.261
261	A	0.591
262	A	0.571
263	A	0.531
264	A	0.306
265	A	0.272
266	A	0.264
267	A	0.351
268	A	0.379
269	A	0.921
270	A	1.58
271	A	3.234
272	A	2.853
273	A	2.014
274	A	1.057
275	A	0.59
276	A	0.628
277	A	0.548
278	A	0.522
279	A	0.507
280	A	0.685
281	A	0.573
282	A	0.596
283	A	1.033
284	A	1.168
285	A	2.65
286	A	4.404

Supplementary Table S5: RMSF Values Profile of SERPINI1

Residue No.	*Chain*	*RMSF Value*
22	A	2.19
23	A	0.755
24	A	0.603
25	A	0.52
26	A	0.332
27	A	0.324
28	A	0.402
29	A	0.389
30	A	0.13
31	A	0.475
32	A	0.511
33	A	0.327
34	A	0.247
35	A	0.253
36	A	0.372
37	A	0.347
38	A	0.478
39	A	0.554
40	A	0.685
41	A	0.92
42	A	1.231
43	A	1.321
44	A	1.71
45	A	0.763
46	A	0.201
47	A	0.326
48	A	0.348
49	A	0.44
50	A	0.376
51	A	0.344
52	A	0.079
53	A	0.157
54	A	0.121
55	A	0.139
56	A	0.354
57	A	0.093
58	A	0.385
59	A	0.459
60	A	0.353
61	A	0.369
62	A	0.437
63	A	0.483

64	A	0.799
65	A	1.175
66	A	1.244
67	A	0.779
68	A	0.34
69	A	0.261
70	A	0.362
71	A	0.189
72	A	0.233
73	A	0.303
74	A	0.188
75	A	0.471
76	A	0.569
77	A	0.62
78	A	0.881
79	A	1.39
84	A	3.288
85	A	1.439
86	A	0.969
87	A	0.533
88	A	0.419
89	A	0.464
90	A	0.327
91	A	0.257
92	A	0.315
93	A	0.514
94	A	0.316
95	A	0.435
96	A	0.673
97	A	0.926
98	A	0.781
99	A	2.456
101	A	2.763
102	A	1.134
103	A	0.743
104	A	0.622
105	A	0.492
106	A	0.31
107	A	0.483
108	A	0.368
109	A	0.445
110	A	0.378
111	A	0.085
112	A	0.101
113	A	0.234
114	A	0.414

115	A	0.931
116	A	0.858
117	A	1.568
118	A	1.277
119	A	1.166
120	A	0.971
121	A	1.807
122	A	1.396
123	A	0.992
124	A	0.826
125	A	0.92
126	A	0.769
127	A	0.626
128	A	0.582
129	A	0.715
130	A	0.773
131	A	0.92
132	A	0.426
133	A	0.578
134	A	0.421
135	A	0.28
136	A	0.36
137	A	0.346
138	A	0.624
139	A	0.728
140	A	0.862
141	A	1.845
142	A	1.433
143	A	1.334
144	A	0.851
145	A	0.845
146	A	0.815
147	A	0.882
148	A	0.65
149	A	0.512
150	A	0.609
151	A	0.679
152	A	0.724
153	A	0.738
154	A	0.873
155	A	1.093
156	A	1.062
157	A	1.13
158	A	1.478
159	A	1.718
160	A	2.869

161	A	2.156
162	A	2.13
163	A	1.812
164	A	1.733
165	A	1.646
166	A	1.069
167	A	0.77
168	A	1.083
169	A	1.097
170	A	2.134
171	A	1.056
172	A	0.837
173	A	1.44
174	A	3.155
175	A	3.152
176	A	0.908
177	A	0.089
178	A	0.199
179	A	0.077
180	A	0.157
181	A	0.297
182	A	0.132
183	A	0.377
184	A	0.456
185	A	0.342
186	A	0.36
187	A	0.482
188	A	0.342
189	A	0.469
190	A	0.343
191	A	0.654
192	A	1.232
193	A	2.003
194	A	2.33
195	A	1.946
196	A	2.221
197	A	2.408
198	A	1.719
199	A	1.814
200	A	0.678
201	A	0.531
202	A	0.622
203	A	0.611
204	A	0.417
205	A	0.66
206	A	0.893

207	A	1.687
208	A	1.627
209	A	1.539
210	A	1.214
211	A	0.767
212	A	0.501
213	A	0.658
214	A	0.423
215	A	0.353
216	A	0.305
217	A	0.303
218	A	0.376
219	A	0.37
220	A	0.426
221	A	0.56
222	A	0.616
223	A	0.292
224	A	0.33
225	A	0.188
226	A	0.387
227	A	0.571
228	A	0.6
229	A	0.778
230	A	1.482
231	A	3.342
237	A	3.402
238	A	0.918
239	A	0.212
240	A	0.142
241	A	0.106
242	A	0.192
243	A	0.184
244	A	0.314
245	A	0.397
246	A	0.777
247	A	1.713
248	A	1.785
249	A	1.708
250	A	0.957
251	A	0.578
252	A	0.347
253	A	0.392
254	A	0.082
255	A	0.082
256	A	0.153
257	A	0.13

258	A	0.641
259	A	1.217
260	A	1.848
261	A	1.887
262	A	1.128
263	A	1.243
264	A	0.943
265	A	1.4
266	A	1.109
267	A	0.946
268	A	1.212
269	A	0.989
270	A	0.944
271	A	0.672
272	A	0.678
273	A	0.362
274	A	0.395
275	A	0.373
276	A	0.372
277	A	0.34
278	A	0.324
279	A	0.325
280	A	0.359
281	A	0.495
282	A	0.506
283	A	0.92
284	A	0.713
285	A	0.591
286	A	0.513
287	A	0.47
288	A	0.444
289	A	0.43
290	A	0.292
291	A	0.337
292	A	0.596
293	A	0.258
294	A	0.446
295	A	0.14
296	A	0.087
297	A	0.122
298	A	0.113
299	A	0.366
300	A	0.089
301	A	0.249
302	A	0.162
303	A	0.197

304	A	0.382
305	A	0.433
306	A	0.49
307	A	0.176
308	A	0.402
309	A	0.541
310	A	0.551
311	A	1.025
312	A	0.804
313	A	0.86
314	A	0.771
315	A	0.777
316	A	0.355
317	A	0.367
318	A	0.695
319	A	0.738
320	A	0.654
321	A	0.999
322	A	0.724
323	A	1.22
324	A	0.781
325	A	0.546
326	A	0.701
327	A	1.262
328	A	1.985
329	A	1.256
330	A	0.729
331	A	0.35
332	A	0.302
333	A	0.145
334	A	0.473
335	A	0.132
336	A	0.161
337	A	0.107
338	A	0.135
339	A	0.06
340	A	0.083
341	A	0.191
342	A	0.059
343	A	0.081
344	A	0.257
345	A	0.917
346	A	0.832
347	A	1.122
348	A	0.78
349	A	1.564

350	A	3.037
351	A	4.743
352	A	6.709
353	A	8.807
359	A	3.664
360	A	3.459
361	A	4.019
362	A	3.85
363	A	4.227
364	A	3.129
365	A	2.44
366	A	2.599
367	A	1.501
368	A	1.291
369	A	0.792
370	A	0.657
371	A	1.474
372	A	0.544
373	A	0.583
374	A	0.533
375	A	0.44
376	A	0.317
377	A	0.253
378	A	0.414
379	A	0.431
380	A	0.284
381	A	0.368
382	A	0.453
383	A	0.723
384	A	0.807
385	A	1.139
386	A	0.899
387	A	0.589
388	A	0.298
389	A	0.233
390	A	0.094
391	A	0.078
392	A	0.34
393	A	0.315
394	A	0.438
395	A	0.775
396	A	1.518
397	A	2.197
398	A	2.663
399	A	2.599
400	A	3.84

Supplementary Table S6: RMSF Values Profile of OPCML

Residue No.	*Chain*	*RMSF Value*
42	A	2.688
43	A	2.498
44	A	2.453
45	A	2.449
46	A	2.416
47	A	2.429
48	A	2.42
49	A	2.44
50	A	2.496
51	A	2.507
52	A	2.53
53	A	2.51
54	A	2.527
55	A	2.533
56	A	2.647
57	A	2.8
58	A	2.892
59	A	3.146
60	A	3.282
61	A	3.551
62	A	3.735
63	A	3.181
64	A	2.926
65	A	2.585
66	A	2.502
67	A	2.447
68	A	2.402
69	A	2.432
70	A	2.417
71	A	2.593
72	A	2.815
73	A	2.562
74	A	2.48
75	A	2.447
76	A	2.478
77	A	2.487
78	A	2.563
79	A	3.067
80	A	2.882
81	A	2.7
82	A	2.771

83	A	2.875
84	A	2.734
85	A	2.755
86	A	2.785
87	A	2.693
88	A	2.625
89	A	2.719
90	A	2.641
91	A	2.702
92	A	2.539
93	A	2.737
94	A	2.8
95	A	3.267
96	A	2.804
97	A	2.726
98	A	2.604
99	A	2.491
100	A	2.593
101	A	2.578
102	A	2.555
103	A	2.586
104	A	2.698
105	A	2.56
106	A	2.901
107	A	3.835
108	A	4.058
109	A	2.912
110	A	2.564
111	A	2.464
112	A	2.466
113	A	2.623
114	A	2.663
115	A	2.508
116	A	2.652
117	A	2.608
118	A	2.778
119	A	3.031
120	A	3.446
121	A	3.472
122	A	3.107
123	A	2.935
124	A	2.829
125	A	2.712
126	A	2.607
127	A	2.496
128	A	2.516

129	A	2.482
130	A	2.456
131	A	2.413
132	A	2.404
133	A	2.377
134	A	2.374
135	A	2.36
136	A	2.34
137	A	2.359
138	A	2.362
139	A	2.41
140	A	2.431
141	A	2.397
142	A	2.426
143	A	2.473
144	A	2.481
145	A	2.506
146	A	2.532
147	A	2.543
148	A	2.564
149	A	2.537
150	A	2.902
151	A	2.762
152	A	2.604
153	A	2.544
154	A	2.433
155	A	2.389
156	A	2.381
157	A	2.349
158	A	2.362
159	A	2.356
160	A	2.358
161	A	2.344
162	A	2.349
163	A	2.34
164	A	2.331
165	A	2.58
166	A	2.566
167	A	2.297
168	A	2.305
169	A	2.318
170	A	2.34
171	A	2.376
172	A	2.826
173	A	4.649
179	A	3.06

180	A	1.369
181	A	1.156
182	A	1.249
183	A	1.62
184	A	2.537
185	A	2.94
186	A	1.815
187	A	1.946
188	A	1.877
189	A	2.018
190	A	1.963
191	A	2.078
192	A	2.543
193	A	2.335
194	A	2.332
195	A	2.319
196	A	2.306
197	A	2.315
198	A	2.321
199	A	2.323
200	A	2.312
201	A	2.324
202	A	2.335
203	A	2.35
204	A	2.375
205	A	2.395
206	A	2.432
207	A	2.452
208	A	2.471
209	A	2.488
210	A	2.456
211	A	2.409
212	A	2.405
213	A	2.382
214	A	2.379
215	A	2.358
216	A	2.366
217	A	2.355
218	A	2.344
219	A	2.363
220	A	2.362
221	A	2.401
222	A	2.404
223	A	2.444
224	A	2.442
225	A	2.439

226	A	2.458
227	A	2.404
228	A	2.454
229	A	2.511
230	A	2.627
231	A	2.734
232	A	2.798
233	A	2.914
234	A	3.04
235	A	3.389
236	A	3.888
237	A	4.024
238	A	3.686
239	A	3.104
240	A	2.858
241	A	2.742
242	A	2.782
243	A	2.671
244	A	2.558
245	A	2.488
246	A	2.422
247	A	2.428
248	A	2.423
249	A	2.422
250	A	2.471
251	A	2.493
252	A	2.519
253	A	2.582
254	A	2.618
255	A	2.664
256	A	2.73
257	A	2.679
258	A	2.763
259	A	2.824
260	A	3.183
261	A	2.858
262	A	2.897
263	A	2.994
264	A	3.311
265	A	3.372
266	A	3.588
267	A	3.659
268	A	3.273
269	A	3.16
270	A	2.911
271	A	2.871

272	A	2.829
273	A	2.808
274	A	2.735
275	A	2.845
276	A	2.871
277	A	2.604
278	A	2.609
279	A	2.619
280	A	2.718
281	A	2.755
282	A	2.757
283	A	2.823
284	A	2.876
285	A	2.96
286	A	3.08
287	A	3.235
288	A	3.299
289	A	3.221
290	A	3.07
291	A	3.011
292	A	2.864
293	A	2.736
294	A	2.677
295	A	2.674
296	A	2.535
297	A	2.634
298	A	2.534
299	A	2.555
300	A	2.536
301	A	2.556
302	A	2.52
303	A	2.521
304	A	2.53
305	A	2.515
306	A	2.618
307	A	2.606
308	A	2.632
309	A	2.633
310	A	2.676
311	A	2.788
312	A	2.918
313	A	3.196
314	A	3.61
315	A	4.084
316	A	4.046
317	A	3.587

318	A	3.586
319	A	3.868

Supplementary Table S7: RMSF Values Profile of LGI1

Residue No.	*Chain*	*RMSF Value*
224	A	1.617
225	A	0.75
226	A	0.573
227	A	0.505
228	A	0.546
229	A	0.723
230	A	0.519
231	A	0.638
232	A	0.515
233	A	0.545
234	A	0.831
235	A	0.677
236	A	0.436
237	A	0.432
238	A	0.14
239	A	0.13
240	A	0.304
241	A	0.247
242	A	0.35
243	A	0.762
244	A	1.51
245	A	2.215
246	A	2.457
247	A	1.04
248	A	0.429
249	A	0.264
250	A	0.186
251	A	0.172
252	A	0.163
253	A	0.319
254	A	0.499
255	A	0.697
256	A	1.322
257	A	1.109
258	A	0.673
259	A	0.446
260	A	0.43
261	A	0.339

262	A	0.463
263	A	0.291
264	A	0.485
265	A	0.711
266	A	1.212
267	A	1.952
268	A	1.963
269	A	2.04
270	A	2.05
271	A	1.4
272	A	0.786
273	A	0.367
274	A	0.515
275	A	0.485
276	A	0.224
277	A	0.598
278	A	0.587
279	A	0.59
280	A	0.767
281	A	0.749
282	A	0.678
283	A	0.486
284	A	0.368
285	A	0.323
286	A	0.291
287	A	0.182
288	A	0.261
289	A	0.429
290	A	0.615
291	A	0.769
292	A	1.526
293	A	1.355
294	A	0.74
295	A	0.502
296	A	0.571
297	A	0.316
298	A	0.333
299	A	0.227
300	A	0.227
301	A	0.573
302	A	0.789
303	A	1.191
304	A	1.393
305	A	0.866
306	A	0.598
307	A	0.483

308	A	0.308
309	A	0.162
310	A	0.229
311	A	0.817
312	A	1.197
313	A	1.696
314	A	2.284
315	A	2.945
316	A	1.85
317	A	1.275
318	A	0.718
319	A	0.689
320	A	0.578
321	A	0.76
322	A	0.662
323	A	0.816
324	A	1.213
325	A	2.301
326	A	2.742
327	A	2.934
328	A	2.717
329	A	1.781
330	A	0.937
331	A	0.645
332	A	0.398
333	A	0.495
334	A	0.4
335	A	0.084
336	A	0.195
337	A	0.417
338	A	0.545
339	A	0.645
340	A	0.592
341	A	1.059
342	A	1.371
343	A	0.522
344	A	0.442
345	A	0.402
346	A	0.199
347	A	0.131
348	A	0.099
349	A	0.125
350	A	0.185
351	A	0.602
352	A	0.848
353	A	1.234

354	A	1.327
355	A	0.989
356	A	0.506
357	A	0.292
358	A	0.146
359	A	0.236
360	A	0.229
361	A	0.313
362	A	0.479
363	A	0.94
364	A	1.469
365	A	1.308
366	A	1.698
367	A	0.774
368	A	0.531
369	A	0.624
370	A	0.643
371	A	0.236
372	A	0.63
373	A	0.434
374	A	0.867
375	A	1.749
376	A	1.348
377	A	0.79
378	A	0.801
379	A	0.449
380	A	0.101
381	A	0.078
382	A	0.329
383	A	0.281
384	A	0.392
385	A	0.393
386	A	0.858
387	A	1.461
388	A	2.242
389	A	3.024
390	A	3.747
391	A	4.614
392	A	4.553
393	A	3.547
394	A	2.564
395	A	1.76
396	A	1.148
397	A	0.634
398	A	0.183
399	A	0.256

400	A	0.263
401	A	0.289
402	A	0.113
403	A	0.762
404	A	1.103
405	A	1.775
406	A	1.076
407	A	0.998
408	A	0.621
409	A	0.405
410	A	0.415
411	A	0.494
412	A	0.192
413	A	0.144
414	A	0.907
415	A	1.522
416	A	2.309
417	A	1.44
418	A	0.723
419	A	0.508
420	A	0.545
421	A	1.206
422	A	0.475
423	A	0.666
424	A	0.645
425	A	0.663
426	A	0.757
427	A	1.362
428	A	1.402
429	A	1.18
430	A	1.013
431	A	1.12
432	A	0.734
433	A	0.409
434	A	0.102
435	A	0.119
436	A	0.072
437	A	0.086
438	A	0.096
439	A	0.151
440	A	0.47
441	A	0.895
442	A	1.503
443	A	0.744
444	A	0.52
445	A	0.285

446	A	0.088
447	A	0.349
448	A	0.101
449	A	0.143
450	A	0.35
451	A	0.524
452	A	0.668
453	A	0.812
454	A	0.571
455	A	0.579
456	A	0.624
457	A	0.264
458	A	0.12
459	A	0.259
460	A	0.517
461	A	1.437
462	A	2.345
463	A	2.525
464	A	2.01
465	A	1.063
466	A	0.434
467	A	0.516
468	A	0.537
469	A	0.904
470	A	0.976
471	A	0.499
472	A	0.536
473	A	0.63
474	A	0.694
475	A	0.677
476	A	0.665
477	A	0.583
478	A	0.166
479	A	0.301
480	A	0.309
481	A	0.107
482	A	0.088
483	A	0.19
484	A	0.49
485	A	0.889
486	A	1.06
487	A	0.263
488	A	0.222
489	A	0.094
490	A	0.081
491	A	0.357

492	A	0.498
493	A	0.887
494	A	0.969
495	A	1.136
496	A	2.11
497	A	2.034
498	A	0.821
499	A	0.73
500	A	0.447
501	A	0.286
502	A	0.099
503	A	0.32
504	A	0.286
505	A	0.335
506	A	0.769
507	A	0.937
508	A	0.852
509	A	0.888
510	A	0.441
511	A	0.171
512	A	0.293
513	A	0.468
514	A	0.356
515	A	0.296
516	A	0.467
517	A	0.641
518	A	0.79
519	A	0.55
520	A	0.842
521	A	0.507
522	A	0.221
523	A	0.06
524	A	0.052
525	A	0.183
526	A	0.225
527	A	0.231
528	A	0.359
529	A	0.391
530	A	0.658
531	A	1.23
532	A	1.26
533	A	0.646
534	A	0.285
535	A	0.313
536	A	0.084
537	A	0.093

538	A	0.062
539	A	0.057
540	A	0.081
541	A	0.583
542	A	0.783
543	A	0.87
544	A	0.705
545	A	0.461
546	A	0.215
547	A	0.185
548	A	0.157
549	A	0.32
550	A	0.381
551	A	1.05

Supplementary Table S8: RMSF Values Profile of CNTN

Residue No.	*Chain*	*RMSF Value*
4	A	3.365
5	A	1.337
6	A	0.713
7	A	0.27
8	A	0.424
9	A	0.383
10	A	0.763
11	A	1.194
12	A	1.772
13	A	1.383
14	A	0.924
15	A	0.561
16	A	0.348
17	A	0.331
18	A	0.306
19	A	0.498
20	A	0.567
21	A	1.298
22	A	2.27
23	A	1.746
24	A	1.86
25	A	1.323
26	A	0.987
27	A	0.655
28	A	0.744
29	A	0.687
30	A	0.534

31	A	0.619
32	A	0.693
33	A	0.507
34	A	0.411
35	A	0.653
36	A	1.004
37	A	1.378
38	A	1.546
39	A	0.969
40	A	0.653
41	A	0.47
42	A	0.626
43	A	0.409
44	A	0.45
45	A	0.744
46	A	1.937
47	A	1.841
48	A	1.144
49	A	0.696
50	A	1.172
51	A	2.269
52	A	2.321
53	A	2.663
54	A	2.831
55	A	3.115
56	A	3.312
57	A	2.103
58	A	1.18
59	A	0.876
60	A	0.66
61	A	0.756
62	A	0.992
63	A	0.855
64	A	0.584
65	A	0.601
66	A	0.842
67	A	0.716
68	A	0.979
69	A	1.728
70	A	1.729
71	A	1.949
72	A	2.029
73	A	1.809
74	A	1.648
75	A	2.063
76	A	0.814

77	A	0.631
78	A	0.141
79	A	0.088
80	A	0.22
81	A	0.21
82	A	0.368
83	A	0.394
84	A	0.459
85	A	0.768
86	A	1.306
87	A	1.699
88	A	0.469
89	A	0.361
90	A	0.206
91	A	0.448
92	A	0.63
93	A	0.493
94	A	0.351
95	A	0.179
96	A	0.127
97	A	0.143
98	A	0.275
99	A	0.264
100	A	0.213
101	A	0.201
102	A	0.183
103	A	0.196
104	A	0.498
105	A	0.572
106	A	0.819
107	A	0.803
108	A	1.242
109	A	1.497
110	A	0.866
111	A	1.619
112	A	0.774
113	A	0.401
114	A	0.592
115	A	0.684
116	A	0.762
117	A	1.448
118	A	0.778
119	A	0.684
120	A	0.289
121	A	0.153
122	A	0.278

123	A	0.337
124	A	0.776
125	A	0.621
126	A	0.559
127	A	0.591
128	A	0.611
129	A	0.204
130	A	0.097
131	A	0.095
132	A	0.185
133	A	0.313
134	A	0.243
135	A	0.278
136	A	0.285
137	A	0.205
138	A	0.081
139	A	0.254
140	A	0.233
141	A	0.381
142	A	1.177
143	A	1.507
144	A	0.794
145	A	0.47
146	A	0.376
147	A	0.288
148	A	0.432
149	A	0.827
150	A	0.924
151	A	0.951
152	A	0.396
153	A	0.224
154	A	0.17
155	A	0.208
156	A	0.667
157	A	0.706
158	A	0.759
159	A	0.724
160	A	1.369
161	A	0.449
162	A	0.388
163	A	0.177
164	A	0.443
165	A	0.42
166	A	0.444
167	A	1.574
168	A	2.589

169	A	2.649
170	A	1.813
171	A	1.98
172	A	0.889
173	A	0.647
174	A	0.232
175	A	0.105
176	A	0.174
177	A	0.204
178	A	0.291
179	A	0.179
180	A	0.108
181	A	0.159
182	A	0.155
183	A	0.31
184	A	1.081
185	A	0.98
186	A	0.825
187	A	0.452
188	A	0.182
189	A	0.291
190	A	0.262
191	A	0.29
192	A	0.383
193	A	0.478
194	A	0.618
195	A	0.541
196	A	0.444
197	A	0.566
198	A	0.683
199	A	0.863
200	A	1.722
201	A	2.825
202	A	3.052
203	A	2.738
204	A	2.066
205	A	1.389
206	A	1.302
207	A	0.991
208	A	0.667
209	A	0.454
210	A	0.469
211	A	0.388
212	A	0.491
213	A	0.296
214	A	0.397

215	A	0.736
216	A	1.296
217	A	0.88
218	A	0.795
219	A	0.698
220	A	0.698
221	A	0.631
222	A	0.86
223	A	1.741
224	A	1.952
225	A	1.382
226	A	1.056
227	A	0.758
228	A	0.541
229	A	0.367
230	A	0.223
231	A	0.118
232	A	0.127
233	A	0.126
234	A	0.412
235	A	0.425
236	A	0.623
237	A	0.696
238	A	0.763
239	A	0.737
240	A	0.511
241	A	0.424
242	A	0.498
243	A	0.469
244	A	0.51
245	A	0.355
246	A	0.792
257	A	3.02
258	A	1.113
259	A	0.563
260	A	0.537
261	A	0.57
262	A	0.731
263	A	0.896
264	A	1.386
265	A	1.558
266	A	1.77
267	A	2.177
268	A	2.267
269	A	2.572
270	A	1.656

271	A	0.999
272	A	0.582
273	A	0.468
274	A	0.505
275	A	0.466
276	A	0.402
277	A	0.393
278	A	0.256
279	A	0.361
280	A	0.45
281	A	1.779
282	A	1.948
283	A	0.714
284	A	0.498
285	A	0.39
286	A	0.354
287	A	0.56
288	A	0.543
289	A	0.499
290	A	0.519
291	A	0.605
292	A	0.547
293	A	0.562
294	A	0.591
295	A	0.721
296	A	0.783
297	A	1.036
298	A	0.507
299	A	0.378
300	A	1.179
301	A	1.03
302	A	0.985
303	A	1.856
304	A	0.825
305	A	0.776
306	A	0.624
307	A	0.677
308	A	0.892
309	A	1.241
310	A	1.235
311	A	1.676
312	A	1.647
313	A	1.424
314	A	1.023
315	A	0.725
316	A	0.405

317	A	0.589
318	A	0.793
319	A	1.209
320	A	1.302
321	A	1.676
322	A	2.72
323	A	2.269
324	A	2.258
325	A	1.473
326	A	0.935
327	A	0.413
328	A	0.358
329	A	0.185
330	A	0.458
331	A	0.25
332	A	0.368
333	A	0.693
334	A	1.31
335	A	0.553
336	A	0.544
337	A	0.779
338	A	1.772
339	A	2.541
340	A	1.735
341	A	1.493
342	A	0.91
343	A	0.733
344	A	0.664
345	A	0.946
346	A	1.133
347	A	1.712
348	A	1.242
349	A	0.875
350	A	0.566
351	A	0.378
352	A	0.417
353	A	0.806
354	A	0.984
355	A	0.795
356	A	1.11
357	A	1.095
358	A	1.412
359	A	1.367
360	A	0.902
361	A	0.576
362	A	0.249

363	A	0.119
364	A	0.104
365	A	0.127
366	A	0.119
367	A	0.248
368	A	0.353
369	A	0.872
370	A	1.601
371	A	1.662
372	A	1.081
373	A	0.555
374	A	0.316
375	A	0.363
376	A	0.153
377	A	0.139
378	A	0.322
379	A	0.292
380	A	0.34
381	A	0.456
382	A	0.757
383	A	1.016
384	A	1.39

Supplementary Table S9: RMSF Values Profile of LY6H

Residue No.	*Chain*	*RMSF Value*
26	A	1.35
27	A	0.678
28	A	0.631
29	A	1.273
30	A	0.714
31	A	0.678
32	A	0.986
33	A	1.253
34	A	1.317
35	A	1.595
36	A	1.695
37	A	1.822
38	A	1.887
39	A	1.648
40	A	1.408
41	A	1.276
42	A	1.29
43	A	0.956

44	A	2.208
45	A	1.49
46	A	1.815
47	A	2.201
48	A	2.071
49	A	1.519
50	A	0.564
51	A	0.332
52	A	0.304
53	A	0.2
54	A	0.209
55	A	0.235
56	A	0.297
57	A	0.398
58	A	0.731
59	A	1.487
60	A	2.489
61	A	3.511
62	A	4.217
63	A	4.168
64	A	2.805
65	A	1.784
66	A	1.28
67	A	0.892
68	A	0.633
69	A	0.386
70	A	0.284
71	A	0.243
72	A	0.343
73	A	0.467
74	A	0.63
75	A	0.956
76	A	2.298
77	A	1.976
78	A	2.341
79	A	2.069
80	A	2.941
81	A	2.933
82	A	2.311
83	A	3.156
84	A	3.683
85	A	3.161
86	A	3.479
87	A	4.582
88	A	4.767
89	A	4.843

90	A	5.31
91	A	6.15
92	A	5.773
93	A	5.484
94	A	6.196
95	A	5.713
96	A	4.009
97	A	2.815
98	A	1.873
99	A	1.146
100	A	0.709
101	A	0.665
102	A	0.531
103	A	0.491
104	A	0.536
105	A	0.65
106	A	1.792
107	A	2.789
108	A	2.992
109	A	2.06
110	A	1.862
111	A	2.787
112	A	3.674
